# Independent Dual-Channel Approach to Mesoscopic Graphene Transistors

**DOI:** 10.3390/nano12183223

**Published:** 2022-09-16

**Authors:** Fernando Sánchez, Vicenta Sánchez, Chumin Wang

**Affiliations:** 1 Instituto de Investigaciones en Materiales, Universidad Nacional Autónoma de México, Mexico City 04510, Mexico; 2 Departamento de Física, Facultad de Ciencias, Universidad Nacional Autónoma de México, Mexico City 04510, Mexico

**Keywords:** independent dual-channel method, graphene field-effect transistor, edge reconstruction defects, linear dislocations, quantum capacitance

## Abstract

Graphene field-effect transistors (GFETs) exhibit unique switch and sensing features. In this article, GFETs are investigated within the tight-binding formalism, including quantum capacitance correction, where the graphene ribbons with reconstructed armchair edges are mapped into a set of independent dual channels through a unitary transformation. A new transfer matrix method is further developed to analyze the electron transport in each dual channel under a back gate voltage, while the electronic density of states of graphene ribbons with transversal dislocations are calculated using the retarded Green’s function and a novel real-space renormalization method. The Landauer electrical conductance obtained from these transfer matrices was confirmed by the Kubo–Greenwood formula, and the numerical results for the limiting cases were verified on the basis of analytical results. Finally, the size- and gate-voltage-dependent source-drain currents in GFETs are calculated, whose results are compared with the experimental data.

## 1. Introduction

Graphene is a one-atom-thick planar sheet of carbon atoms in a honeycomb structure, whose free-standing monolayer was isolated and characterized for the first time in 2004 by Andre Geim and Konstantin Novoselov [1]. This material possesses outstanding properties such as high specific surface area [2], very high carrier mobility with null effective mass [3], huge thermal conductivity, and giant Young’s modulus [4], making it an ideal candidate for a wide range of applications [5].

When the semiconductor layer of a traditional metal–oxide–semiconductor field-effect transistor (MOSFET) is replaced by a graphene sheet, it is called as graphene field-effect transistor (GFET), whose source-drain current is extremely sensitive to the adsorbed molecules, and it is employable for use in gas and bio sensing [6,7,8]. Moreover, GFET constitutes a central component of far-infrared radiation and terahertz devices [9] since graphene exhibits a quick optical response based on high carrier mobility [10]. In addition, the graphene nanoribbon field-effect transistor (GNRFET) presents an elevated $I_{on}/I_{off}$ ratio around the threshold voltage compared to the conventional Si-based CMOS [11]. Among possible disadvantages of GFETs, there are difficulties in fabricating wafer-sized high-quality graphene, as well as the apparent degradation of carrier mobility when it is placed over an oxide [12].

Additionally, GFET has a singular capacitor behavior, since the graphene ribbon, being one of the parallel plates, possesses a very low charge density around the Dirac point, which may induce large variations in its chemical potential when a small amount of electric charge is introduced into the capacitor. This fact could significantly increase the potential difference between the back gate and the graphene, because it is equal to the difference between the chemical potentials at each plate. Such small quantities of charge generating a large potential difference lead to a lower capacitance compared to geometric or classic ones ($C_{C}$) obtained using ideal metal plates. Hence, the capacitance of GFET $\left(C_{tot}\right)$ can be calculated by including a so-called quantum capacitance ($C_{Q}$) through $C_{tot}^{-1}=C_{C}^{-1}+C_{Q}^{-1}$ [13].

On the theoretical side, GFETs can be modeled at the atomic scale by means of first-principle or semiempirical methods. For example, N/B/P co-doped seven-atom-wide armchair GNRFET was analyzed by using the density functional theory (DFT) [14], while ten-atom-width GNRFET has been studied by self-consistently solving Schrödinger–Poison equations within the tight-binding formalism [15]. Recently, mesoscopic GFETs have been extensively investigated for the detection of micron-scale biomolecules [16] and their graphene ribbons contain several billions of atoms, which impede atomic-scale first-principle modeling. In addition, such graphene ribbons usually possess numerous structural defects, which requires an innovative real-space approach [17].

In this article, the source-drain current ($I_{sd}$) in mesoscopic GFETs is studied by means of an independent dual-channel method that transforms the graphene ribbon with edge reconstruction defects and transversal dislocations into a set of double chains with first and second neighbor interactions within the tight-binding formalism. The electrical conductance and electronic density of states in these double chains or dual channels connected to two semi-infinite periodic leads at their ends are further addressed by means of a new transfer matrix method within the Landauer formalism and a novel real-space renormalization procedure based on the retarded Green’s function, respectively. The theoretical $I_{sd}$, as a function of the gate voltage, is compared with experimental data obtained from GFETs of different lengths.

## 2. The Model

**Figure 1:**
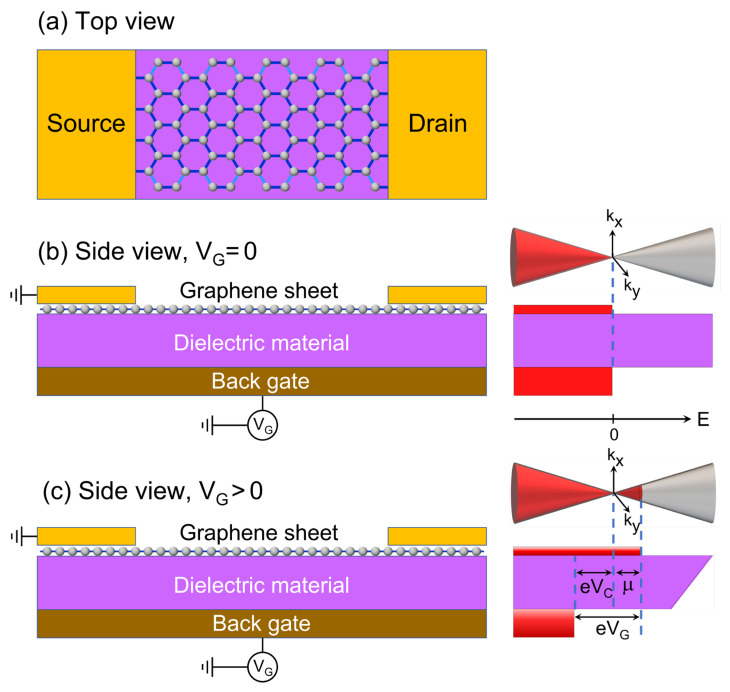
Schematic representations of (**a**) top and (**b**,**c**) side views of a backgated GFET with Dirac cones and energy band diagrams for (**b**) *V_G_* = 0 and (**c**) *V_G_* > 0, where *eV_G_* = *eV_C_* + *µ* being *µ* the chemical potential of graphene ribbon with respect to the Dirac point.

In GFETs, an extra carrier density ($n_{2D}$) can be induced in graphene ribbon by the application of a gate voltage ($V_{G}$), which is related to the chemical potentials of graphene (μ) and of back gate metal ($\mu_{M}$) through $eV_{G}=\mu -\mu_{M}$. Figure 1 shows a schematic representation of (Figure 1a) top and (Figure 1b,c) side views of a backgated GFET with energy band diagrams for (b) $V_{G}=0$ and (c) $V_{G}>0$, where the diminution of $\mu_{M}$ due to $V_{G}$ is denoted as $eV_{C}$. Hence, we have:

(1) $$eV_{G}=\mu -\mu_{M}=\mu +eV_{C},$$

which is equivalent to $C_{tot}^{-1}=C_{C}^{-1}+C_{Q}^{-1}$, since $C_{tot}=e\;\partial n/\partial V_{G}$, $C_{C}=e\;\partial n/\partial V_{C}$, $C_{Q}=e\;\partial n/\partial V_{Q}$ and $e\;V_{Q}=\mu -\mu_{0}$ with $\mu_{0}=0$ or at the Dirac point in our case [18].

In general, the capacitance per unit of area (*C*) of an ideal parallel-plate capacitor with a dielectric material of thickness *d* and a relative permittivity $\varepsilon_{r}$ is $C=\varepsilon_{r}\varepsilon_{0}/d=Q/V_{C}$ in units of MKS [19], where $\varepsilon_{0}$ is the vacuum permittivity, $Q=en_{2D}$ is the electric charge per unit of area in the capacitor and $V_{C}$ is the potential difference between the parallel plates. Hence:

(2) $$eV_{C}=\frac{e^{2}n_{2D}d}{\varepsilon_{r}\varepsilon_{0}},$$

where *e* is the magnitude of the electron charge.

**Figure 2:**
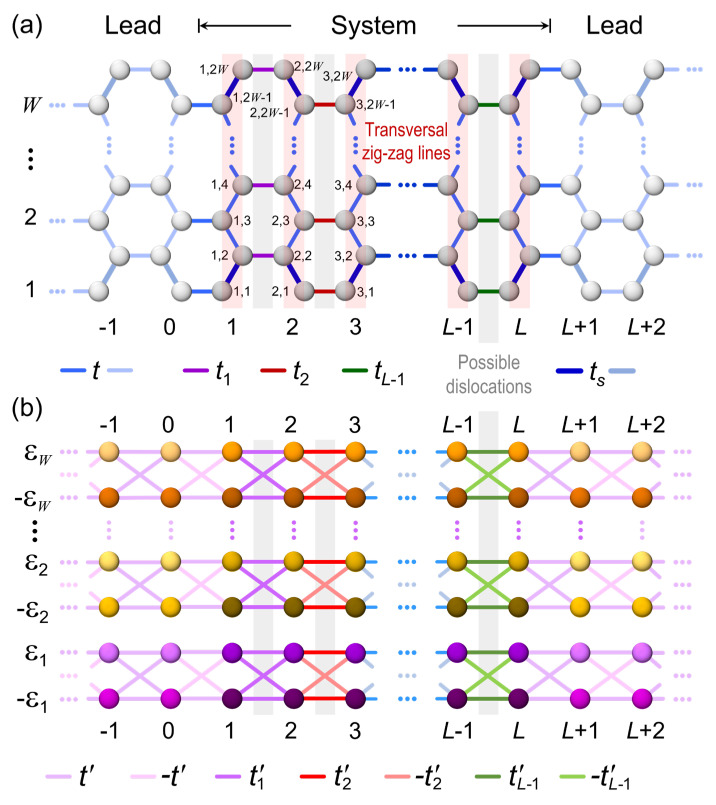
Sketch of (**a**) a graphene ribbon of width *W* and length *L* with hopping integrals *t*, $t_{s}$ and $t_{l}$, being l=1, 2, ⋯, L−1, connected to two semi-infinite periodic leads (light grey spheres) and (**b**) double chains obtained from the independent dual-channel method (Appendix A) with hopping integrals $\pm \;t^{\prime }$, $\pm \;{t^{\prime }}_{l}$ and on-site energies $\pm \;\varepsilon_{j}$ being j=1, 2,⋯, W. Red highlight and gray zones respectively indicate zig-zag lines and possible transversal dislocations.

Let us consider an armchair-edged graphene ribbon with an even number of atoms (*2W*) in each transversal zig-zag line, as illustrated in Figure 2a. This ribbon of width $W\;=\sqrt{3}a_{S}+\sqrt{3}(2W-3)\;a_{0}/2$ and length $L=(3/2)La_{0}-a_{0}$ is found in a backgated GFET, where *W* and *L* are integer numbers, $a_{0}=1.41$ Å and $a_{S}=1.36$ Å are bond lengths between interior atoms and between the edge and next-edge atoms, respectively denoted by blue and thick blue lines in Figure 2a. This variation in interatomic distance is originated from the edge reconstruction of graphene ribbons [20]. The single-electron tight-binding Hamiltonian (*H*) of this graphene ribbon with null on-site energies can be written as

(3) $$\begin{array}{cc} H= & \sum_{l\;=\;1}^{L}\left(t_{s}|l,1\rangle \langle l,2|+t\sum_{j\;=\;2}^{2W-2}|l,j\rangle \langle l,j+1|+t_{s}|l,2W-1\rangle \langle l,2W|\right)\\ \;+ & \sum_{l\;=\;1}^{\left(L-1\right)/2}\sum_{j\;=\;1}^{W}\left(t_{2l-1}|2l-1,2j\rangle \langle 2l,2j|+t_{2l}|2l,2j-1\rangle \langle 2l+1,2j-1|\right), \end{array}$$

where $|l,j\rangle$ is the Wannier function at site (l,j), *t* is the hopping integral of pristine graphene sheet and $t_{s}\;=1.2\;t$ is that between the edge and next-edge atoms in a graphene ribbon with edge reconstructions [20] and $t_{l}$ with l=1, 2, ⋯, L−1 is the hopping integral between transversal zig-zag lines *l* and l+1, representing possible transversal dislocations when $t_{l}\;\neq t$.

There is a unitary transformation discussed in Appendix A that converts the Hamiltonian (3), including the edge reconstruction defects, into a set of *W* independent dual channels, whose Hamiltonian contains hopping integrals $t^{\prime }\;=t/2$, ${t^{\prime }}_{l}\;=t_{l}/2$ with l=1, 2, ⋯, L−1 and on-site energies $\pm \;\varepsilon_{j}$ with j=1, 2,⋯, W, which are eigenvalues obtained from the Hamiltonian of an arbitrary transversal zig-zag line in Figure 2.

The extra carrier density ($n_{2D}$) induced by $V_{G}$ in the graphene ribbon can be calculated by [21]

(4) $$n_{2D}=\int_{\;0}^{\mu }DOS(E)\;dE=\int_{0}^{eV_{G}-\;eV_{C}}DOS(E)\;dE,$$

where

(5) DOS(E)=−1πImTr [G+(E)],

is the density of states with $G^{+}(E)\;=\underset{\eta \;\to \;0^{+}}{\lim}{\left(E+i\eta -H\right)}^{-1}$ the retarded Green’s function and *η* the imaginary part of energy [22]. Combining Equations (2) and (4), $V_{C}$ can be determined as the solution of following self-consistent equation,

(6) $$eV_{C}\;=\frac{e^{2}d}{\varepsilon_{r}\varepsilon_{0}}\int_{0}^{eV_{G}-\;eV_{C}}DOS(E)\;dE.$$

On the other hand, the source-drain current ($I_{sd}$) of GFETs at temperature *T* is given by [23]

(7) $$I_{sd}(T)=V_{sd}g(\mu ,T),$$

where $V_{sd}$ is the source-drain voltage and

(8) $$g(\mu ,T)=\int_{-\infty }^{+\infty }dE\left(-\frac{\partial f_{FD}(E)}{\partial E}\right)\;g(E,0)=g_{0}\int_{-\infty }^{+\infty }dE\left(-\frac{\partial f_{FD}(E)}{\partial E}\right)\;T(E)$$

is the electrical conductance of graphene ribbon at chemical potential *μ* within the Landauer formalism. In Equation (8), $f_{FD}={\left\{\exp[\left(E-\mu \right)/k_{B}T]+1\right\}}^{-1}$ is the Fermi–Dirac distribution, $g_{0}=2e^{2}/h$ is the conductance quantum, T(E) is the carrier transmittance of graphene ribbon, and at zero temperature we have [24]

(9) $$g(\mu ,0)=g_{0}T(\mu ).$$

By means of the independent dual-channel method presented in Appendix A, an armchair-edged graphene ribbon can be transformed into a set of double chains or dual channels (see Figure 2) and then

(10) $$T(E)=\sum_{j=1}^{W}T_{j}(E),$$

where $T_{j}(E)$ is the carrier transmittance along the *j*-th dual channel. We further developed a new transfer matrix method to calculate $T_{j}(E)$, which is thoroughly presented in Appendix B, and the result can be written as

(11) $$T_{j}(E)={\left|\frac{u_{0}(j)\;d_{0}^{*}(j)-d_{0}(j)\;u_{0}^{*}(j)}{m_{11}(j,E)\;d_{0}(j)\;u_{0}^{*}(j)+m_{12}(j,E)|d_{0}(j)\;{|}^{2}-\;m_{21}(j,E)\;|u_{0}(j)\;{|}^{2}-\;m_{22}(j,E)\;u_{0}(j)\;d_{0}^{*}(j)}\right|}^{2},$$

where $m_{\mu \nu }(j,E)$ are elements of the total transfer matrix, $u_{0}(j)$ and $d_{0}(j)$ are respectively initial wavefunction amplitudes at the up and down channels, both from the *j*-th dual channel (see Appendix B).

One of structural defects commonly observed in graphene ribbons is linear dislocation [25,26], because its presence slightly alters the free energy but may significantly modify the carrier transport. In this article, we consider a distribution of transversal dislocations following the Rudin–Shapiro (RS) sequence, which constitutes one of the closest aperiodic arrays to the random distribution [27]. A Rudin–Shapiro lattice with bond disorder can be built by using four kinds of dislocation hopping integrals $t_{l}=t_{A},\;t_{B},\;t_{C}\;\mathrm{or}\;t_{D}$, respectively denoted by letters *A*, *B*, *C* or *D*, whose positions along the ribbon are ordered by the substitution rules [28]

(12) A→A⊕B=AB, B→A⊕C=AC, C→D⊕B=DB, D→D⊕C=DC

or by the addition rules expressed as

(13) $$\begin{array}{l} S_{A}(k+1)=S_{A}(k)⊕S_{B}(k),\;S_{B}(k+1)=S_{A}(k)⊕S_{C}(k),\;\\ S_{C}(k+1)=\;S_{D}(k)⊕S_{B}(k),\;S_{D}(k+1)=\;S_{D}(k)⊕S_{C}(k), \end{array}$$

where the symbol ⊕ represents the concatenation operation and $S_{\alpha }(k)$ is the *α*-type RS sequence of generation *k* with α= A,B,C or D. As a consequence, a graphene ribbon based on $S_{\alpha }(k)$ has $2^{k}+1$ transversal zig-zag lines. To have a limited number of dislocations, let us introduce dilute RS lattices, which are built by using periodic segments to construct the RS lattice until generation m−1 and a single transversal dislocation is placed at the initial of next generation *m*. For example, when k=m=3, $S_{A}(3)=APPP$, $S_{B}(3)=BPPP$, $S_{C}(3)=CPPP$ and $S_{D}(3)=DPPP$, where *P* denotes a periodic RS sequence with hopping integral *t* connecting all transversal zig-zag lines. A detailed discussion of this dilute RS lattice and the real-space renormalization method can be found in Appendix C.

Another quantity that should be considered for determining the electrical conductance is the contact resistance between the graphene ribbon and periodic leads. In the diffusive regime, the total resistance ($R_{total}$) can be written as [29,30]

(14) $$R_{total}=R+2R_{c}/W,$$

where $R=R_{sheet}L/W$ is the electrical resistance of a graphene ribbon with length L and width W, being $R_{sheet}$ that of a square graphene sheet, and $R_{c}$ is the specific contact resistance in units of Ω · μm, which can be experimentally determined by the transfer length method [31]. Hence, the total conductance defined as $G_{total}=R_{total}^{-1}$ can be calculated from Equation (14) through

(15) $$G_{total}=\frac{G}{2(R_{c}/W)\;G+1},$$

where $G=R^{-1}$ is the conductance of graphene ribbon. For a uniform sample, the electrical resistance of a *p*-micron segment, *R*(L=p μm), should be p/q times of *R*(L=q μm) obtained from a *q* micrometer portion. Therefore, Equation (15) can be rewritten as

(16) $$G_{total}(p\;\mu m)=\frac{G(p\;\mu m)}{2(R_{c}/W)\;G(p\;\mu m)+1}=\frac{G(q\;\mu m)}{2(R_{c}/W)\;G(q\;\mu m)+p/q},$$

where G(p μm)=qG(q μm)/p. This equation will be used in the length variation analysis.

## 3. Results

The electronic density of states, $DOS^{\alpha }(k,E)$, of a graphene ribbon built by *W* dual channels based on an *α*-type dilute RS sequence of generation *k* can be written as [22]

(17) DOSα(k,E)=∑j=1WDOSjα(k,E)=∑j=1W−1πlimη → 0+∑l=1L(k)Im [Gl,lα,j(k,z)],

where $L(k)=2\;(2^{k}+1)$ is the number of sites in each dual channel and $G_{l,l}^{\alpha ,j}(k,z)$ is the *l*-th diagonal element of the retarded Green’s function evaluated at z=E+iη. The $DOS_{j}^{\alpha }(k,E)$ of the *j*-th dual channel can be rewritten as

(18) DOSjα(k,E)=−1πlimη → 0+Im Mα(k) GL,Lα,j(k,z) +Pα(k) GL_,L_α,j(k,z) + Qα(k) GR,Rα,j(k,z)      + Rα(k) GR_,R_α,j(k,z) +Sα(k) GL,L_α,j(k,z)+Uα(k) GL,Rα,j(k,z)+Vα(k) GL,R_α,j(k,z)     + Wα(k) GL_,Rα,j(k,z)+ Xα(k) GL_,R_α,j(k,z)+Yα(k) GR,R_α,j(k,z)+Zα(k),

where $M^{\alpha }(k)\;,\;\cdots \;,\;Z^{\alpha }(k)$ are the renormalization coefficients and $G_{\mu ,\nu }^{\alpha ,j}(k,z)$ with $\mu ,\nu =L,\;\underline{L},\;R\;\mathrm{or}\;\underline{R}$ are matrix elements of the Green’s function corresponding to the left up (*L*), left down (*L*), right up (*R*) and right down (*R*) sites of the renormalized four-site *j*-th dual channel, which constitutes the basic element of the real-space renormalization method presented in Appendix C.

**Figure 3:**
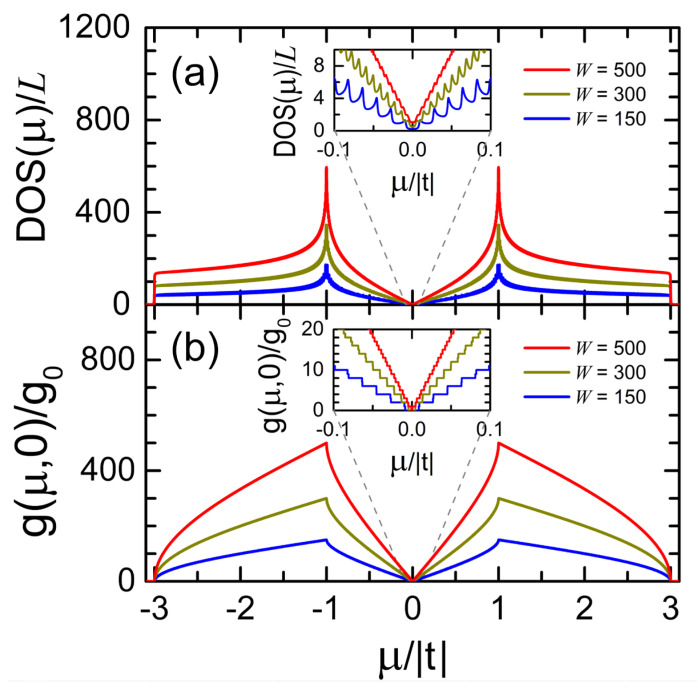
(**a**) Electronic density of states (DOS) and (**b**) electrical conductance (g) at T=0 in units of $g_{0}$ versus the chemical potential (μ) for graphene ribbons of W=0.122 μm (red lines), 0.073 μm (dark yellow lines), and 0.037 μm (blue lines) with an arbitrary length of *L* transversal zig-zag lines and hopping integrals of *t* and $t_{s}=1.2\;t$.

Figure 3 shows the electronic density of states (DOS) and the electrical conductance (g) at zero temperature in units of the conductance quantum $g_{0}=2e^{2}/h$ obtained from Equation (9) as functions of the chemical potential (μ) for free-standing graphene ribbons without dislocations, whose widths are W=0.122  μm (red lines), 0.073  μm (dark yellow lines) and 0.037  μm (blue lines) as well as an arbitrary length of *L* transversal zig-zag lines. All the ribbons studied in this article are connected to two semi-infinite periodic leads at their ends, as illustrated in Figure 2a, while a surface hopping integral $t_{s}=1.2\;t$ due to the edge reconstruction and an imaginary part of energy $\eta =10^{-3}|\;t\;|$ are also included.

Observe the Van Hove singularities in the inset of Figure 3a, which are located at the band limits of each dual channel. The DOS spectra of Figure 3a can be analytically verified through [32]

(19) $$DOS(E)=\frac{dS/dk}{|dE/dk\;|}=\sum_{j=\;1}^{W}\frac{2|E|}{\pi \sqrt{4t^{2}\varepsilon_{j}^{2}-{\left(E^{2}-4t^{2}-\varepsilon_{j}^{2}\right)}^{2}}},$$

where dS/dk=La/(2π) and dE/dk can be obtained from Equation (A9) in Appendix B. Note also the quantized conductance spectra in the inset of Figure 3b and the maximum values of g(E,0) around E=± t in Figure 3b is equal to the number of dual channels (*W*).

**Figure 4:**
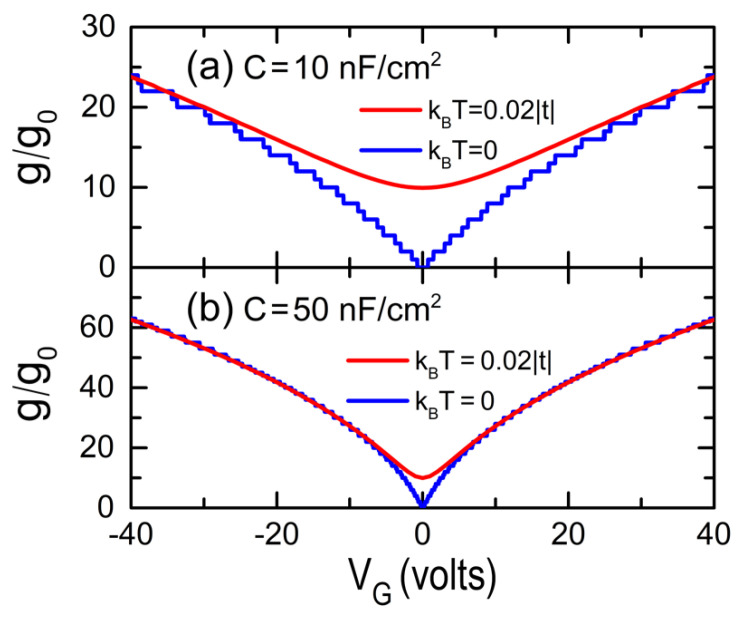
Normalized electrical conductance (g) by the conductance quantum $g_{0}$ at T=0 and at $k_{B}T=0.02|\;t\;|$ as a function of the gate voltage $\left(V_{G}\right)$ for GFETs with (**a**) $C=10\;\mathrm{nF}/{\mathrm{cm}}^{2}$ and (**b**) $C=50\;\mathrm{nF}/{\mathrm{cm}}^{2}$, whose dimensions are W=0.122 μm and L=4097 zig-zag lines without dislocations.

To analyze the electrical conductance (g) of GFETs, we first self-consistently calculate $V_{C}$ from Equation (6) for a given $V_{G}$ and then, the chemical potential μ and g can be respectively determined using Equations (1) and (8). Figure 4 shows the electrical conductance (g) at T =0 (blue lines) and at $k_{B}T=0.02|\;t\;|$ (red lines) versus the gate voltage ($V_{G}$) for GFETs of W=0.122 μm, i.e., W=500 dual channels, and L=4097 zig-zag lines without dislocations, whose capacitances per unit area are (a) $C=10\;\mathrm{nF}/{\mathrm{cm}}^{2}$ and (b) $C=50\;\mathrm{nF}/{\mathrm{cm}}^{2}$. Note the quantized conductance spectra for T =0, in contrast to the smoothed ones when *T* increases, as well as the growth of conductance with the capacitance.

**Figure 5:**
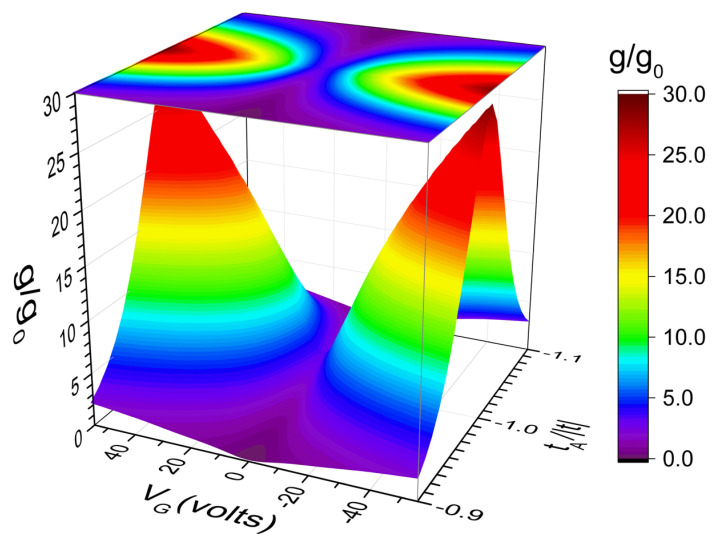
Electrical conductance (g) at $k_{B}T\;=0.026|\;t\;|$ as a function of gate voltage ($V_{G}$) and dislocation hopping integral $t_{A}$ for the same GFET of Figure 4a except by 72 *A*-type transversal dislocations lines placed following the dilute RS sequence with m=5 and $t_{B}=t_{C}=t_{D}=t$.

In Figure 5, the electrical conductance (g) at $k_{B}T\;=0.026|\;t\;|$ is plotted as a function of both the back gate voltage $\left(V_{G}\right)$ and the dislocation hopping integral $t_{A}$ for the same graphene ribbon of Figure 4 except by the value of $t_{A}$ maintaining $t_{B}=t_{C}=t_{D}=t$. This ribbon contains 72 *A*-type transversal dislocation lines obtained from a *A*-type dilute RS lattice with k=12 and dislocations introduced from generation m=5, and it is placed on a GFET with a capacitance per unit area $C=10\;\mathrm{nF}/{\mathrm{cm}}^{2}$. Observe the V-shaped dependence between g and $V_{G}$, even for $t_{A}=0.9\;t$, and this dependence can be noted from Figure 3b, since $V_{G}$ modifies the value of chemical potential μ, as shown in Figure 1c.

**Figure 6:**
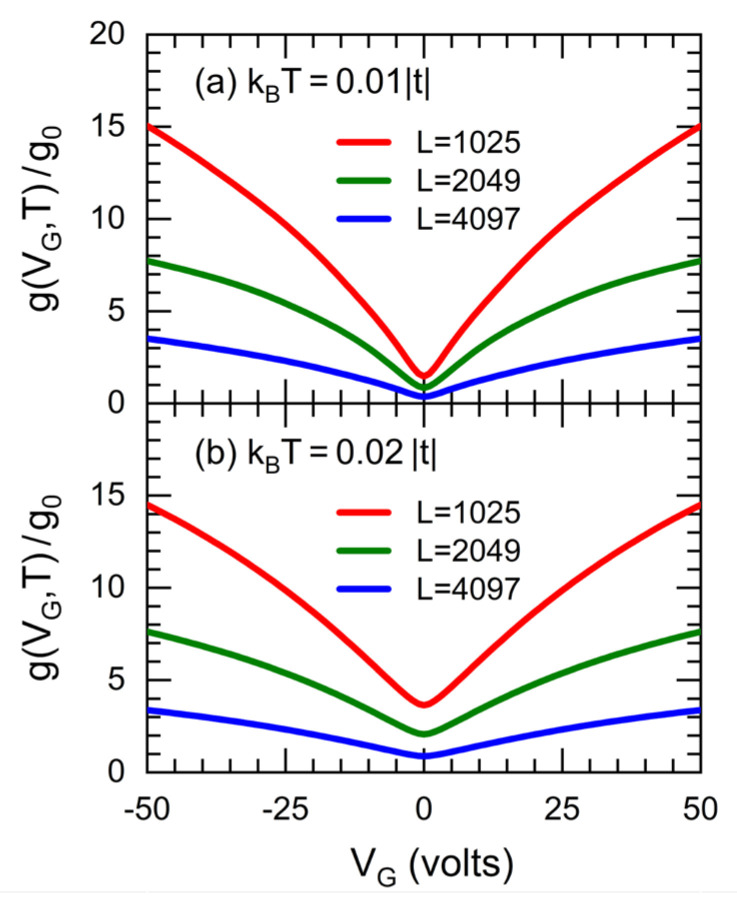
Conductance (g) at (**a**) $k_{B}T=0.01|\;t\;|$ and (**b**) $k_{B}T=0.02|\;t\;|$ as functions of the gate voltage ($V_{G}$) for GFETs with dislocations and a capacitance $C=10\;\mathrm{nF}/{\mathrm{cm}}^{2}$, W=0.122 μm (i.e., W=500 dual channels) and L=0.217 μm (red lines), 0.433 μm (green lines), and 0.866 μm (blue lines), whose hopping integrals are $t_{A}=-2.389\;\mathrm{eV}$, $t_{B}=t_{C}=-2.74\;\mathrm{eV}$ and $t_{D}=-2.63\;\mathrm{eV}$.

Figure 6 shows the electrical conductance (g) at (Figure 6a) $k_{B}T=0.01|t|$ and (Figure 6b) $k_{B}T=0.02|t|$ with t=−2.70 eV [20] as functions of the gate voltage ($V_{G}$) for GFETs of W=0.122 μm (i.e., W=500 dual channels) and L=0.217 μm (red lines), 0.433 μm (green lines), and 0.866 μm (blue lines), respectively corresponding to generations k=10, 11 and 12 of *A*-type dilute RS sequences with dislocations introduced from generation m=3, hopping integrals $t_{A}=-2.389\;\mathrm{eV}$, $t_{B}=t_{C}=-2.74\;\mathrm{eV}$ and $t_{D}=-2.63\;\mathrm{eV}$, where the capacitance per unit area of GFET is $C=10\;\mathrm{nF}/{\mathrm{cm}}^{2}$. 

The results reveal a more emphasized V-shaped conductance behavior at lower temperatures, while the electrical conductance diminishes with increasing RS generation, being almost inversely proportional to the GFET length, in accordance with the Ohm’s law as well as the length variation measurements carried out in GFET [33].

**Figure 7:**
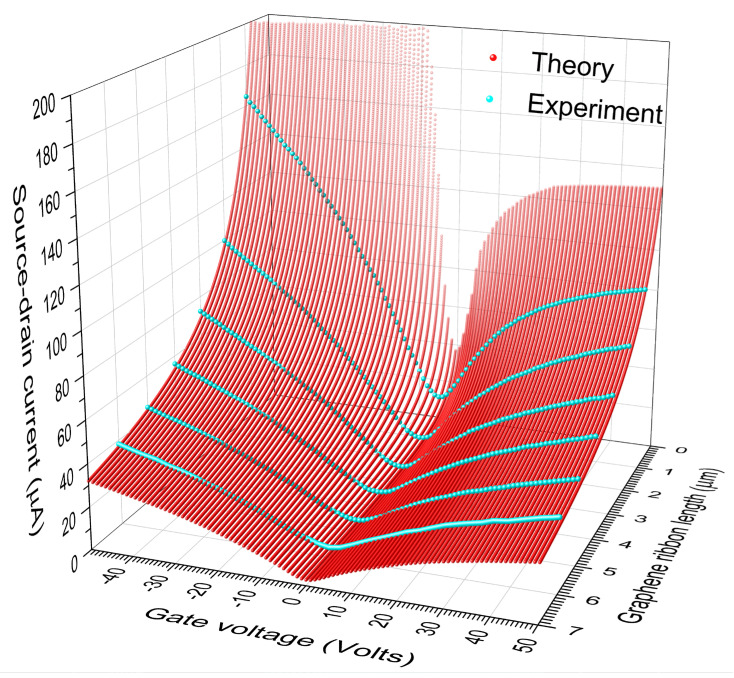
Theoretical source-drain current (red spheres) versus gate voltage ($V_{G}$) and graphene ribbon length (L) for a GFET of a width W=2.2 μm and a capacitance per unit area $C=12.11\;\mathrm{nF}/{\mathrm{cm}}^{2}$, in comparison with experimental data (cyan spheres) reported from Ref. [28] for L=1, 2, ⋯, 6 μm.

Finally, let us consider a specific GFET [29] built by an armchair-edged graphene ribbon placed on a substrate of silicon dioxide (SiO_2_) of thickness d=285 nm, i.e., a geometric capacitance per unit area of $C=\varepsilon_{0}\varepsilon_{r}/d\approx 12.11\;\mathrm{nF}/{\mathrm{cm}}^{2}$. We first calculated the electrical conductance g of a graphene ribbon having a width of W =9009 dual channels (W=2.2 μm) and a length of L=4097 zig-zag lines (L=0.866 μm corresponding to generation 12 of the RS sequence), which contains 1024 transversal dislocation lines from a *A*-type dilute RS lattice with m=3 and hopping integrals t=−2.7 eV, $t_{s}=1.2\;t$, $t_{A}=-2.389\;\mathrm{eV}$, $t_{B}=t_{C}=-2.74\;\;\mathrm{eV}$ and $t_{D}=-2.63\;\;\mathrm{eV}$ as in Figure 6, while the analysis of system length (L) effects on the conductance was carried out by means of Equation (16). Additionally, the contact resistance described in ref. [29] is included in the total conductance $\left(G_{total}\right)$ using Equation (15). In Figure 7, we present the source-drain current ($I_{sd}$) at T=300  K as a function of the gate voltage $\left(V_{G}\right)$ and graphene ribbon length (L) for the considered GFET with a source-drain voltage of $V_{sd}\;=0.05\;\;\mathrm{volts}$, where $I_{sd}(T)$ (red spheres) was obtained from $I_{sd}=V_{sd}G_{total}$ and Equation (16) with q=0.866 μm and p=L.

The accordance between the theoretical ($I_{sd}^{T}$) and experimental ($I_{sd}^{E}$) source-drain currents can be quantified by means of the standard deviation (σ), given by

(20) $$\sigma =\sqrt{\frac{1}{N}\sum_{j=1}^{N}{\left\{I_{sd}^{T}[V_{G}(j)]-I_{sd}^{E}[V_{G}(j)]\right\}}^{2}},$$

where $V_{G}(j)=j-51\;\mathrm{volts}$ is the *j*-th analyzed gate voltage and $I_{sd}^{E}[V_{G}(j)]$ are digitalized experimental data (cyan spheres in Figure 7) from reference [29] with N =101. The resulting σ for graphene ribbons of L=1, 2, 3, 4, 5 and 6 μm are respectively 3.4423, 3.0119, 2.5801, 2.7210, 1.9327 and 1.4795 μA.

Note in Figure 7 the asymmetry of $I_{sd}$ values with respect to $V_{G}\;=0$, whose magnitude diminishes as the ribbon length increases. This asymmetry is derived from that of the contact-resistance ($R_{c}$) spectrum in ref. [29], which is probably related to the slight doping of the used graphene ribbon [30]. In fact, the contribution of $R_{c}$ to $I_{sd}$ diminishes as the resistance of graphene ribbon (*R*) grows [see Equation (15)], while *R* in turn increases with the ribbon length. Therefore, the asymmetry of $I_{sd}$ respect to $V_{G}\;=0$ should decrease as the ribbon length grows.

## 4. Conclusions

The correlation between source-drain current and gate voltage in graphene field-effect transistors (GFETs) is investigated by means of an atomic-scale tight-binding model, where mesoscopic graphene ribbons with edge reconstruction defects and transversal dislocations are addressed by an independent dual-channel transformation, which converts armchair-edged graphene ribbons into a set of independent dual channels.

The electronic transport was studied within the Landauer formalism, and a new transfer matrix method has been further developed for each dual channel with nearest- and next-nearest-neighbor interactions, including aperiodically placed dislocations following a dilute Rudin–Shapiro (RS) sequence, which is one of the closest aperiodic arrangements to the random distribution. The Landauer conductance was confirmed by that obtained from the Kubo–Greenwood formula (see Appendix B), while the density of states from the Green’s function has been verified by analytical solutions of Equation (19) for the case without dislocations.

Quantized electrical conductance spectra are observed even in presence of edge reconstruction defects along mesoscopic graphene ribbons without dislocations, whose maximum value corresponds to the number of dual channels, while the multi-step behavior is softened at finite temperatures. In addition, the back gate voltage ($V_{G}$) creates a shift of the chemical potential in graphene ribbons, which produces a V-shape correlation between the source-drain current ($I_{sd}$) and $V_{G}$ originated from the V-shape DOS spectrum of graphene around the Dirac point. Finally, the realistic calculation of $I_{sd}$ reveals an excellent agreement between the theoretical prediction from a single set of parameters and multiple experimental data obtained from GFETs of six different lengths [29].

The approach of independent dual-channel plus renormalization method presented in this article can be used to study biosensor devices based on GFETs by considering adsorbed molecules as Fano impurities [34]. This study is currently in process.

## Figures and Tables

**Figure A1:**
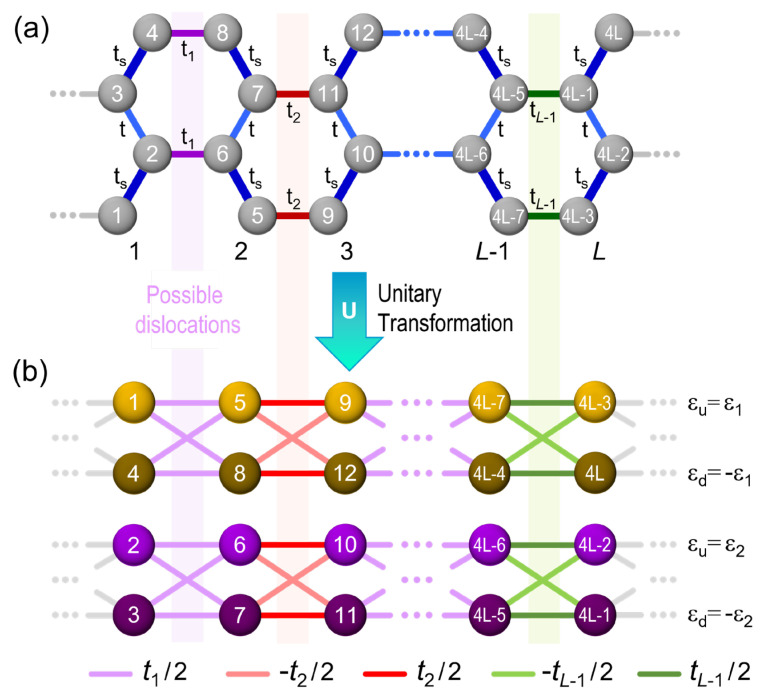
(**a**) Sketch of an armchair-edged graphene nanoribbon with a width of 4 atoms, arbitrary length *L*, null on-site energies, hopping integrals *t* (light blue lines), $t_{1}$ (violet lines), $t_{2}$ (red lines), $t_{L-1}$ (green lines) and $t_{s}=1.2\;t$ (thick blue lines); (**b**) by applying a unitary transformation **U**, the nanoribbon (**a**) becomes into two independent dual-channels with on-site energies ($\pm \varepsilon_{1}$ and $\pm \varepsilon_{2}$) and hopping integrals $t_{1}/2$ (light violet lines), $\pm t_{2}/2$ (light red lines) and $\pm t_{L-1}/2$ (light green lines). Numbers inside spheres denote the positions in Hamiltonian matrices (A1) and (A4) respectively before and after the application of **U**.

**Figure A2:**
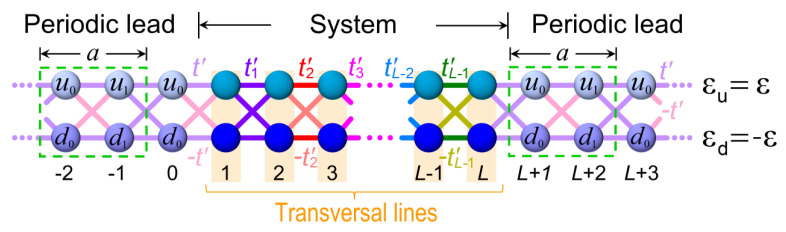
Dual-channel system of 2*L* sites with hopping integrals $\pm \;{t^{\prime }}_{l}$ connected to two semi-infinite periodic leads with $\pm \;t^{\prime },$ where the on-site energies are $\varepsilon_{u}=\varepsilon$ and $\varepsilon_{d}=-\varepsilon$. Green dashed lines indicate unit cells whose wavefunction amplitudes without phases are denoted on the spheres.

**Figure A3:**
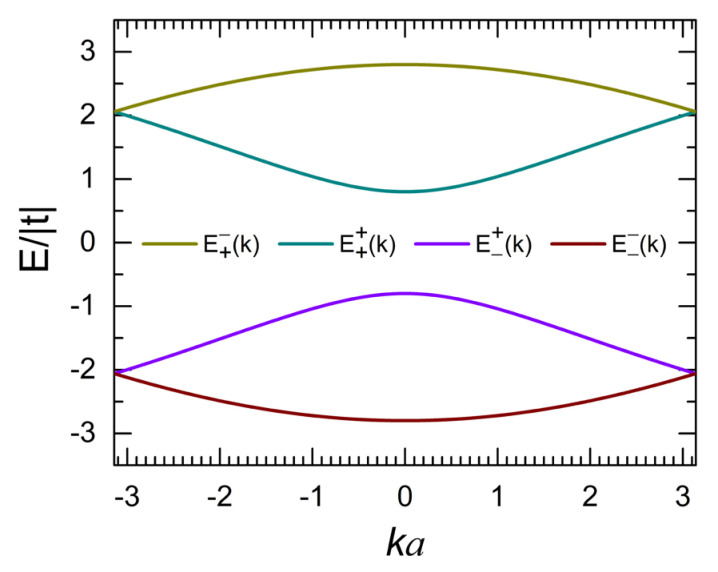
Dispersion relations $E_{\pm }^{+}(k)$ and $E_{\pm }^{-}(k)$ versus *ka* for periodic dual-channel leads with $\varepsilon_{u}=-\;\varepsilon_{d}\;=1.8|\;t\;|$ and $t^{\prime }\;=-\;0.5|\;t\;|$.

**Figure A4:**
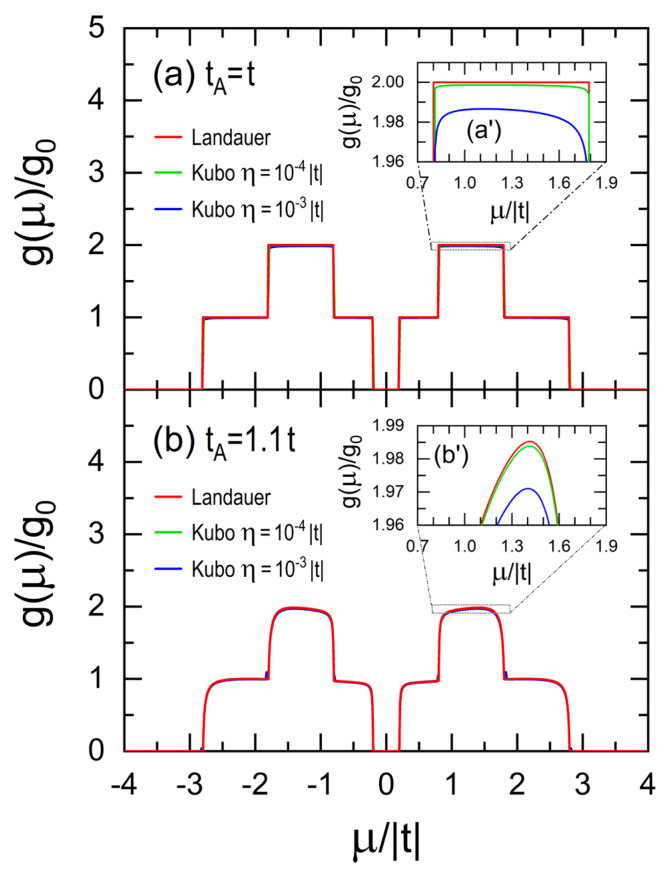
Electrical conductance (g) at 0 K in units of $g_{0}=2e^{2}/h$ obtained from the Landauer (red lines) and Kubo–Greenwood formulas using $\eta =10^{-3}|\;t\;|$ (blue lines) and $\eta =10^{-4}|\;t\;|$ (green lines) as functions of the chemical potential (μ) for graphene ribbons with W=4, L =9, $t_{s}=1.2\;t,$ $t_{B}\;=t$, (**a**,**a**′) $t_{A}=t$ and (**b**,**b**’) $t_{A}=1.1\;t$, whose magnifications are shown in (**a’**) and (**b’**).

**Figure A5:**
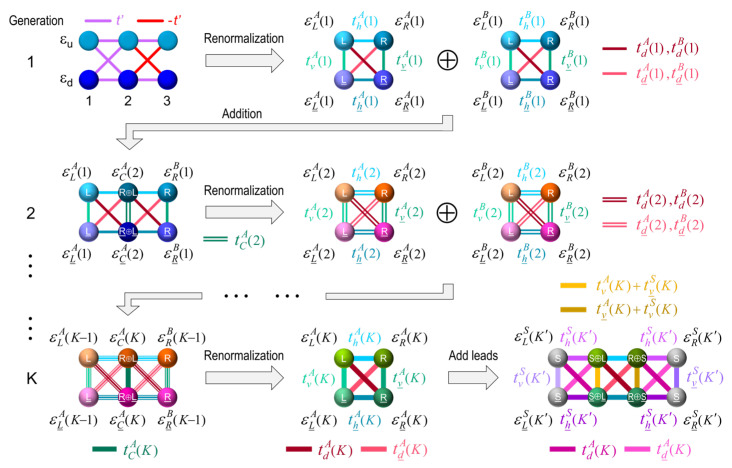
Schematic representation of the renormalization procedure to build an *A*-type RS dual channel of generation *K* connected to two periodic leads of generation *K’* (gray spheres), where the effective on-site energies $\varepsilon_{l}^{\alpha }(k,z)$ and hopping integrals $t_{j}^{\alpha }(k,z)$ are respectively denoted as $\varepsilon_{l}^{\alpha }(k)$ and $t_{j}^{\alpha }(k)$ for α=A or B, $l=L,\;\underline{L},\;C,\;\underline{C},\;R\;\mathrm{or}\;\underline{R}$ and $j=h,\;\underline{h},\;v,\;\underline{v},\;d,\;\underline{d},\;C\;\mathrm{or}\;\underline{C}$.

## Data Availability

Data sharing not applicable.

## Appendix A. Independent Dual-Channel Method

Let us consider an armchair-edged graphene ribbon with a width of four atoms and an arbitrary length of *L* transversal zig-zag lines described by Hamiltonian (3), which in the matrix form following the atom numbering of Figure A1a can be written as

(A1) $$H=\left(\begin{array}{ccccccc} \ddots & \ddots & \ddots & \vdots & ⋰ & \ddots & \ddots \\ \ddots & h & t_{1} & 0 & \cdots & \ddots & \ddots \\ \ddots & t_{1} & h & t_{2} & \ddots & \vdots & ⋰\\ \cdots & 0 & t_{2} & h & \ddots & 0 & \cdots \\ ⋰ & \vdots & \ddots & \ddots & \ddots & t_{L-1} & \ddots \\ \ddots & \ddots & \cdots & 0 & t_{L-1} & h & \ddots \\ \ddots & \ddots & ⋰ & \vdots & \ddots & \ddots & \ddots \end{array}\right),$$

where

(A2) $$h=\left(\begin{array}{cccc} 0 & t_{s} & 0 & 0\\ t_{s} & 0 & t & 0\\ 0 & t & 0 & t_{s}\\ 0 & 0 & t_{s} & 0 \end{array}\right),\;t_{1}=\left(\begin{array}{cccc} 0 & 0 & 0 & 0\\ 0 & t_{1} & 0 & 0\\ 0 & 0 & 0 & 0\\ 0 & 0 & 0 & t_{1} \end{array}\right),\;t_{2}=\left(\begin{array}{cccc} t_{2} & 0 & 0 & 0\\ 0 & 0 & 0 & 0\\ 0 & 0 & t_{2} & 0\\ 0 & 0 & 0 & 0 \end{array}\right),\;\mathrm{and}\;t_{L-1}=\left(\begin{array}{cccc} t_{L-1} & 0 & 0 & 0\\ 0 & 0 & 0 & 0\\ 0 & 0 & t_{L-1} & 0\\ 0 & 0 & 0 & 0 \end{array}\right)$$

being *t* and $t_{s}$ the interior- and surface-hopping integrals, whose difference is originated from the edge reconstruction of graphene ribbons [20]. In Equation (A2), $t_{l}$ with l=1, 2, ⋯, L−1 are hopping integrals that connect zig-zag lines and through them transverse dislocations can be introduced. The eigenvalues of matrix h are $\pm \;\varepsilon_{1}=\pm \frac{1}{2}\left(t-\sqrt{t^{2}+4t_{s}^{2}}\right)$ and $\pm \;\varepsilon_{2}=\mp \frac{1}{2}\left(t+\sqrt{t^{2}+4t_{s}^{2}}\right)$.

Let us further introduce a unitary transformation given by

(A3) $$U=\left(\begin{array}{ccccc} \ddots & \ddots & \ddots & \vdots & ⋰\\ \ddots & u & 0 & 0 & \cdots \\ \ddots & 0 & u & 0 & \ddots \\ \cdots & 0 & 0 & u & \ddots \\ ⋰ & \vdots & \ddots & \ddots & \ddots \end{array}\right)\;\mathrm{with}\;u=\frac{1}{\sqrt{2}}\left(\begin{array}{cccc} \upsilon_{1} & -\;\upsilon_{2} & \upsilon_{2} & -\;\upsilon_{1}\\ \varepsilon_{1}\upsilon_{1}/t_{s} & -\varepsilon_{2}\upsilon_{2}/t_{s} & -\varepsilon_{2}\upsilon_{2}/t_{s} & \varepsilon_{1}\upsilon_{1}/t_{s}\\ \varepsilon_{1}\upsilon_{1}/t_{s} & \varepsilon_{2}\upsilon_{2}/t_{s} & -\varepsilon_{2}\upsilon_{2}/t_{s} & -\varepsilon_{1}\upsilon_{1}/t_{s}\\ \upsilon_{1} & \upsilon_{2} & \upsilon_{2} & \upsilon_{1} \end{array}\right),$$

where $\upsilon_{j}={\left[{\left(\varepsilon_{j}/t_{s}\right)}^{2}+1\right]}^{-1/2}$ being j=1 or 2.

Applying this transformation (**U**) to **H** we obtain

(A4) $$\tilde{H}=U^{T}H\;U=\left(\begin{array}{ccccccc} \ddots & \ddots & \ddots & \vdots & ⋰ & \ddots & \ddots \\ \ddots & \alpha & \beta_{1} & 0 & \cdots & \ddots & \ddots \\ \ddots & \beta_{1} & \alpha & \beta_{2} & \ddots & \vdots & ⋰\\ \cdots & 0 & \beta_{2} & \alpha & \ddots & 0 & \cdots \\ ⋰ & \vdots & \ddots & \ddots & \ddots & \beta_{L-1} & \ddots \\ \ddots & \ddots & \cdots & 0 & \beta_{L-1} & \alpha & \ddots \\ \ddots & \ddots & ⋰ & \vdots & \ddots & \ddots & \ddots \end{array}\right),$$

where

(A5) $$\alpha =\left(\begin{array}{cccc} \varepsilon_{1} & 0 & 0 & 0\\ 0 & \varepsilon_{2} & 0 & 0\\ 0 & 0 & -\varepsilon_{2} & 0\\ 0 & 0 & 0 & -\varepsilon_{1} \end{array}\right),\;\beta_{1}=\left(\begin{array}{cccc} {t^{\prime }}_{1} & 0 & 0 & {t^{\prime }}_{1}\\ 0 & {t^{\prime }}_{1} & {t^{\prime }}_{1} & 0\\ 0 & {t^{\prime }}_{1} & {t^{\prime }}_{1} & 0\\ {t^{\prime }}_{1} & 0 & 0 & {t^{\prime }}_{1} \end{array}\right),\;\beta_{2}=\left(\begin{array}{cccc} {t^{\prime }}_{2} & 0 & 0 & -{t^{\prime }}_{2}\\ 0 & {t^{\prime }}_{2} & -{t^{\prime }}_{2} & 0\\ 0 & -{t^{\prime }}_{2} & {t^{\prime }}_{2} & 0\\ -{t^{\prime }}_{2} & 0 & 0 & {t^{\prime }}_{2} \end{array}\right)\;\mathrm{and}\;\beta_{L-1}=\left(\begin{array}{cccc} {t^{\prime }}_{L-1} & 0 & 0 & -{t^{\prime }}_{L-1}\\ 0 & {t^{\prime }}_{L-1} & -{t^{\prime }}_{L-1} & 0\\ 0 & -{t^{\prime }}_{L-1} & {t^{\prime }}_{L-1} & 0\\ -{t^{\prime }}_{L-1} & 0 & 0 & {t^{\prime }}_{L-1} \end{array}\right)$$

being ${t^{\prime }}_{l}=t_{l}/2$ with l=1, 2, ⋯, L− 1.

Hence, for a graphene ribbon with a width of four atoms and arbitrary length *L*, Hamiltonian H can be visualized as two independent dual channels, as illustrated in Figure A1b with on-site energies $\pm \varepsilon_{j}$ and hopping integrals $t_{1}/2$, $\pm \;t_{2}/2$ and $\pm t_{L-1}/2$. It is worth mentioning that the independent dual-channel method presented in this appendix includes the ribbon edge reconstruction, and a particular case of this method can be found in reference [35] for $t_{s}=t$.

## Appendix B. Transfer Matrix Method for Dual Channels

Let us consider a dual channel or double chain obtained from the unitary transformation of Appendix A, as illustrated in Figure A2, whose Hamiltonian can be written as

(A6) $$\begin{array}{c} H=\varepsilon \sum_{l}\left(|u,l\rangle \langle u,l|-|d,l\rangle \langle d,l|\right)+\sum_{l}{t^{\prime }}_{l}\left(|d,l\rangle \langle d,l+1|+|u,l\rangle \langle u,l+1|+c.c.\right)\\ \;+\;\sum_{l}{t^{\prime }}_{l}\left(|d,2l-1\rangle \langle u,2l|+|u,2l-1\rangle \langle d,2l|-|d,2l\rangle \langle u,2l+1|-|u,2l\rangle \langle d,2l+1|+c.c.\right), \end{array}$$

where ${t^{\prime }}_{l}\;=t_{l}/2$ is the hopping integral between transversal lines *l* and l+1, $|u\;,\;l\rangle$ and $|d,l\rangle$ are respectively the Wannier functions at the up and down sites of transversal line *l*. In contrast, there is no variation in hopping integrals ${t^{\prime }}_{l}\;=t^{\prime }\;=t/2$ in periodic leads. Moreover, the system possesses an odd number *L* of transversal lines, since a Rudin–Shapiro (RS) distribution of bonds is considered.

In these periodic dual-channel leads, the unit cell of length *a* is constituted by four sites and the electronic wavefunction with wavenumber *k* can be written as

(A7) $$|\Psi (k)\rangle =\sum_{m}\left[u_{0}(k)\;|u,0\rangle +u_{1}(k)\;e^{ika/2}|u,1\rangle +d_{0}(k)\;|d,0\rangle +d_{1}(k)\;e^{ika/2}|d,1\rangle \right]\;e^{ikma},$$

where *m* is the index of unit cells. The stationary Schrödinger equation $H|\Psi (k)\rangle =E(k)|\Psi (k)\rangle$ leads to

(A8) $$\left(\begin{array}{cccc} \varepsilon & 0 & 2\;t^{\prime }\cos\;(ka/2) & -2\;i\;t^{\prime }\sin\;(ka/2)\\ 0 & -\varepsilon & -2\;i\;t^{\prime }\sin\;(ka/2) & 2\;t^{\prime }\cos\;(ka/2)\\ 2\;t^{\prime }\cos\;(ka/2) & 2\;i\;t^{\prime }\sin\;(ka/2) & \varepsilon & 0\\ 2\;i\;t^{\prime }\sin\;(ka/2) & 2\;t^{\prime }\cos\;(ka/2) & 0 & -\varepsilon \end{array}\right)\left(\begin{array}{c} u_{0}(k)\\ d_{0}(k)\\ u_{1}(k)\\ d_{1}(k) \end{array}\right)=E(k)\left(\begin{array}{c} u_{0}(k)\\ d_{0}(k)\\ u_{1}(k)\\ d_{1}(k) \end{array}\right),$$

whose eigenvalues or dispersion relations are

(A9) E(k)={E+−(k)=+ε2+4(t′)2−4 tε cos (ka/2), if ε2+4 (t′)2≤E≤ ε−2 t′E++(k)=+ε2+4(t′)2+4 tε cos (ka/2), if ε+2 t′ ≤E≤ε2+4 (t′)2E−+(k)=−ε2+4(t′)2+4 tε cos (ka/2), if −ε2+4 (t′)2≤E≤−ε+2 t′E−−(k)=−ε2+4(t′)2−4 tε cos (ka/2), if − ε−2 t′ ≤E≤−ε2+4 (t′)2

where *a* is the lattice constant indicated in Figure A2. These four dispersion relations of (A9) are plotted in Figure A3 for $t^{\prime }=-\;0.5|\;t\;|$, $t_{s}=-\;1.2|\;t\;|$ and $\varepsilon_{u}=-\varepsilon_{d}=\frac{1}{2}\left(|\;t\;|+\sqrt{t^{2}+4t_{s}^{2}}\right)=1.8\;|\;t\;|$ in the first dual channel analyzed in Appendix A.

Wavefunction amplitudes $u_{l}(k)$ and $d_{l}(k)$ for l=0 and 1 obtained from Equation (A8) are summarized in Table A1 for each energy band, shown for example in Figure A3.

**Table A1:** Amplitudes $u_{l}$ and $d_{l}$ in periodic dual-channel leads with $\varepsilon_{u}=-\varepsilon_{d}=\varepsilon >0$ and $t^{\prime }<0$.

Energy Band	*u*_0_ (*k*)	*d*_0_ (*k*)	*u*_1_ (*k*)	*d_1_* (*k*)
$\left|\varepsilon +2t^{\prime }\right|\;\leq E\leq \sqrt{\varepsilon^{2}+4{\left(t^{\prime }\right)}^{2}}$	$-i\;[\;C+\varepsilon +E_{+}^{+}]/S$	−1	$-i\;[\;C+\varepsilon +E_{+}^{+}]/S$	1
$-\sqrt{\varepsilon^{2}+4{\left(t^{\prime }\right)}^{2}}\leq E\leq -\left|\varepsilon +2t^{\prime }\right|$	$-i\;[C+\;\varepsilon +E_{-}^{+}]/S$	−1	$-i\;[\;C+\varepsilon +E_{-}^{+}]/S$	1
$\sqrt{\varepsilon^{2}+4{\left(t^{\prime }\right)}^{2}}\leq E\leq \;\left|2t^{\prime }-\varepsilon \right|$	$i\;[\;C-\varepsilon -E_{+}^{-}]/S$	1	$-i\;[\;C-\varepsilon -E_{+}^{-}]/S$	1
$-\;\left|2t^{\prime }-\varepsilon \right|\;\leq E\leq -\sqrt{\varepsilon^{2}+4{\left(t^{\prime }\right)}^{2}}$	$i\;[\;C-\varepsilon -E_{-}^{-}]/S$	1	$-i\;[C-\;\varepsilon -E_{-}^{-}]/S$	1

where $C=2t^{\prime }\cos\;(ka/2)$ and $S=2t^{\prime }\sin\;(ka/2)$.

On the other hand, for a dual-channel system of *L* transversal lines connected to two periodic leads represented by lines zero and L+1, the wavefunction of both system and leads can be written as

(A10) $$|\Psi \rangle =\sum_{l=0}^{L+1}\left(A_{l}|u,l\rangle +B_{l}|d,l\rangle \right)$$

and then, the stationary Schrödinger equation from Hamiltonian (A6) is given by

(A11) $$\left\{ \begin{array}{c} {}[\varepsilon_{u}-E]\;A_{1}+{t^{\prime }}_{0}A_{0}+{t^{\prime }}_{1}A_{2}-{t^{\prime }}_{0}B_{0}+{t^{\prime }}_{1}B_{2}=0\\ {}[\varepsilon_{d}-E]\;B_{1}+{t^{\prime }}_{0}B_{0}+{t^{\prime }}_{1}B_{2}-{t^{\prime }}_{0}A_{0}+{t^{\prime }}_{1}A_{2}=0\\ {}[\varepsilon_{u}-E]\;A_{2}+{t^{\prime }}_{1}A_{1}+{t^{\prime }}_{2}A_{3}+{t^{\prime }}_{1}B_{1}-{t^{\prime }}_{2}B_{3}=0\\ {}[\varepsilon_{d}-E]\;B_{2}+{t^{\prime }}_{1}B_{1}+{t^{\prime }}_{2}B_{3}+{t^{\prime }}_{1}A_{1}-{t^{\prime }}_{2}A_{3}=0\\ \vdots \\ {}[\varepsilon_{u}-E]\;A_{L-1}+{t^{\prime }}_{L-2}A_{L-2}+{t^{\prime }}_{L-1}A_{L}+{t^{\prime }}_{L-2}B_{L-2}-{t^{\prime }}_{L-1}B_{L}=0\\ {}[\varepsilon_{d}-E]\;B_{L-1}+{t^{\prime }}_{L-2}B_{L-2}+{t^{\prime }}_{L-1}B_{L}+{t^{\prime }}_{L-2}A_{L-2}-{t^{\prime }}_{L-1}A_{L}=0\\ {}[\varepsilon_{u}-E]\;A_{L}+{t^{\prime }}_{L-1}A_{L-1}+{t^{\prime }}_{L}A_{L+1}-{t^{\prime }}_{L-1}B_{L-1}+{t^{\prime }}_{L}B_{L+1}=0\\ {}[\varepsilon_{d}-E]\;B_{L}+{t^{\prime }}_{L-1}B_{L-1}+{t^{\prime }}_{L}B_{L+1}-{t^{\prime }}_{L-1}A_{L-1}+{t^{\prime }}_{L}A_{L+1}=0 \end{array},\right.$$

where $\varepsilon_{u}=\varepsilon$, $\varepsilon_{d}=-\varepsilon$ and ${t^{\prime }}_{0}\;={t^{\prime }}_{L}\;=t^{\prime }$. Equations (A11) can be rewritten in matrix form as

(A12) $$\left(\begin{array}{c} A_{L+1}\\ B_{L+1} \end{array}\right)=M_{L}(E)\cdots M_{2j}(E)M_{2j-1}(E)\cdots M_{0}(E)\left(\begin{array}{c} A_{0}\\ B_{0} \end{array}\right)=M_{T}(E)\left(\begin{array}{c} A_{0}\\ B_{0} \end{array}\right)=\left(\begin{array}{cc} m_{11}(E) & m_{12}(E)\\ m_{21}(E) & m_{22}(E) \end{array}\right)\left(\begin{array}{c} A_{0}\\ B_{0} \end{array}\right),$$

where j=1, 2, ⋯ , (L−1)/2, $A_{0}$ ($B_{0}$) and $A_{L+1}$ ($B_{L+1}$) are respectively wavefunction coefficients at the up (down) site of transversal lines zero and L+1,

(A13) $$M_{2l}(E)=\frac{1}{4\;\varepsilon \;t_{2l}}\left(\begin{array}{cc} E^{2}-\varepsilon^{2}-4\;t_{2l}^{2} & -{\left(E+\varepsilon \right)}^{2}+4\;t_{2l}^{2}\\ {\left(E-\varepsilon \right)}^{2}-4\;t_{2l}^{2} & -E^{2}+\varepsilon^{2}+4\;t_{2l}^{2} \end{array}\right)\;\mathrm{for}\;l=0,\;1,\;\cdots \;,\;\frac{L-1}{2}$$

(A14) $$M_{2s-1}(E)=\frac{1}{4\;\varepsilon \;t_{2s-1}}\left(\begin{array}{cc} E^{2}-\varepsilon^{2}-4\;t_{2s-1}^{2} & -{\left(E-\varepsilon \right)}^{2}+4\;t_{2s-1}^{2}\\ -{\left(E-\varepsilon \right)}^{2}+4\;t_{2s-1}^{2} & E^{2}-\varepsilon^{2}-4\;t_{2s-1}^{2} \end{array}\right)\;\mathrm{for}\;s=1,\;2,\;\cdots \;,\;\frac{L+1}{2}$$

and the total transfer matrix $M_{T}$ including connections to periodic leads $M_{L}$ and $M_{0}$.

For the case of an electronic plane wave incident from the left lead of Figure A1, the scattered ones by the system are a reflected wave moving to the left and another transmitted one going to the right. Hence, the wavefunction coefficients at transversal line zero and line L+1 can be respectively written as

(A15) $$\left(\begin{array}{c} A_{0}\\ B_{0} \end{array}\right)=\left(\begin{array}{c} u_{0}+r\;u_{0}^{*}\\ d_{0}+r\;d_{0}^{*} \end{array}\right)\;\mathrm{and}\;\left(\begin{array}{c} A_{L+1}\\ B_{L+1} \end{array}\right)=\left(\begin{array}{c} \tau \;u_{0}e^{ik(L+1)a}\\ \tau \;d_{0}e^{ik(L+1)a} \end{array}\right),$$

where *r* and τ are respectively the reflection and transmission coefficients satisfying $|\;r\;{|}^{2}+|\;\tau \;{|}^{2}=1$. From Equations (A12) and (A15), we have

(A16) $$\left(\begin{array}{c} u_{0}\tau e^{ik(L+1)a}\\ d_{0}\tau e^{ik(L+1)a} \end{array}\right)=M_{T}(E)\left(\begin{array}{c} u_{0}+u_{0}^{*}r\\ d_{0}+d_{0}^{*}r \end{array}\right),$$

which can be rewritten as

(A17) $$\left(\begin{array}{c} \tau e^{ik\;La}\\ 0 \end{array}\right)={\left(\begin{array}{cc} u_{0}e^{ika} & 0\\ d_{0}e^{ika} & 1 \end{array}\right)}^{-1}\left(\begin{array}{cc} m_{11}(E) & m_{12}(E)\\ m_{21}(E) & m_{22}(E) \end{array}\right)\left(\begin{array}{c} u_{0}+r\;u_{0}^{*}\\ d_{0}+r\;d_{0}^{*} \end{array}\right).$$

Therefore, the transmittance (T) of electronic waves in a dual channel is given by

(A18) $$T(E)={\left|\tau \right|}^{2}={\left|\frac{u_{0}\;d_{0}^{*}-d_{0}\;u_{0}^{*}}{m_{11}(E)\;d_{0}u_{0}^{*}+m_{12}(E)\;|d_{0}\;{|}^{2}-\;m_{21}(E)\;|u_{0}\;{|}^{2}-\;m_{22}(E)\;u_{0}\;d_{0}^{*}}\right|}^{2}$$

where $u_{0}$ and $d_{0}$ are given in Table A1.

The electrical conductance (g) at 0 K obtained from Equations (9) and (A18) as a function of the chemical potential (μ) is shown in Figure A4 for the graphene ribbon of Figure A1 containing L=9 transversal lines (see Table A3 for generation k=3) (a) without and (b) with a single transversal dislocation $t_{A}=1.1\;t$, while $t_{B}=\;\;t$. These Landauer conductance spectra are further compared with those obtained from the Kubo formalism within the linear response theory. In general, the electric conductivity (σ) can be calculated by means of the Kubo–Greenwood formula [22],

(A19) σ(μ,ω,T)=2 e2ℏπ m2Ω∫−∞∞dEfFD(E)−fFD(E+ℏω)ℏωTrp^xImG+(z+ℏω)p^xImG+(z),

where *e* and *m* are respectively the electrical charge and the mass of electron, Ω is the area of graphene ribbon, and $f_{FD}(E)={\left\{\;1+\exp\left[(E-\mu )/k_{B}T\right]\;\right\}}^{-1}$ is the Fermi–Dirac distribution with the chemical potential μ, the Boltzmann constant $k_{B}$ and temperature *T*. ${\hat{p}}_{x}\;=im\;[\hat{H},x]/\hbar$ is the projection of the momentum operator along the oscillating electrical field with frequency ω, and $G^{+}(z)$ with z=E+iη is the retarded one-particle Green’s function. For the case of ω=T=0, Equation (A19) can be rewritten as [36].

(A20) g(μ,0,0)=σ(μ,0,0)WL=2 e2ℏπ m2L2Trp^xImG+(μ)p^xImG+(μ).

In Figure A4 the Kubo conductance spectra are obtained from Equation (A20) for two imaginary parts of energy $\eta =10^{-3}|\;t\;|$ (blue lines) and $\eta =10^{-4}|\;t\;|$ (green lines), where the retarded Green’s function was calculated through $G(z)={\left(z-H\right)}^{-1}$ from the Dyson equation for a Hamiltonian of 36 atoms since W =4 and L=9 for a dilute *A*-type RS lattice with k=4 and m=3 containing only dislocation hopping integrals $t_{A}$ and $t_{B}$, whose boundary conditions were obtained from periodic leads of 200,000 transversal lines. Observe in Figure A4a’,b’ the coincidence between electrical conductance spectra from Landauer and Kubo formalisms when η→0 in the latter one.

## Appendix C. Real-Space Renormalization Method for Dilute Rudin–Shapiro Dual Channels

The Rudin–Shapiro (RS) sequence of generation *k*, S(k), can be built using the substitution rules (12) or addition rules (13). The latter can be summarized into a single equation given by

(A21) $$S_{\alpha }(k)=S_{\beta }(k-1)⊕S_{\gamma }(k-1),$$

whose subindices *α*, *β* and *γ* denote *A*, *B*, *C* or *D*-type RS sequence according to Table A2.

**Table A2:** Addition rules for all type Rudin–Shapiro sequences.

*α*-Type	*β*-Type	*γ*-Type
*A*	*A*	*B*
*B*	*A*	*C*
*C*	*D*	*B*
*D*	*D*	*C*

Let us consider an RS dual channel with bond disorder formed by alternating hopping integrals ${t^{\prime }}_{A},$ ${t^{\prime }}_{B},$ ${t^{\prime }}_{C}$ and ${t^{\prime }}_{D}$, instead of letters *A*, *B*, *C* and *D* in the RS sequences, being ${t^{\prime }}_{\alpha }=t_{\alpha }/2$ with α=A, B, C or D. Moreover, a dilute RS dual channel can be built using periodic RS segments of generation m−1 formed by hopping integrals $t^{\prime }=t/2$ and placing a single hopping integral defect ${t^{\prime }}_{\alpha }$ at the beginning of $S_{\alpha }(m)$, while subsequent generations of dilute RS dual channels are constructed following the standard addition rule (A21) and Table A2. For example, dilute RS dual channels with m=2 are presented in Table A3.

**Table A3:** Dilute Rudin–Shapiro dual channels with *m* = 2.

Generation	*A*-Type	*B*-Type	*C*-Type	*D*-Type
1				
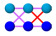
2				
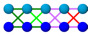
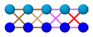
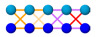
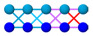
3				
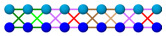
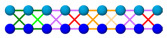
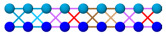
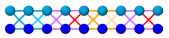

Note: Symbol meaning .

In this appendix, a new real-space renormalization method is developed for dilute RS dual channels, as an extension of that for single channels developed in reference [37], where the freedom degrees of interior sites are removed but their exact participation is maintained in the results. As an example, we present in Figure A5 this renormalization procedure for *A*-type RS dual channels by starting from generation one, which consists of six sites (blue spheres) with on-site energies $\varepsilon_{u}\;=\varepsilon$ and $\varepsilon_{d}\;=-\;\varepsilon$, respectively, for up and down sites, as well as hopping integrals $t^{\prime }=t/2$ (violet lines) and $-\;t^{\prime }$ (red lines). The central sites located at the second transversal line can be renormalized by reducing the six-site dual channel into a four-site one with effective on-site energies $\varepsilon_{L}^{A}(1)$, $\varepsilon_{\underline{L}}^{A}(1)$, $\varepsilon_{R}^{A}(1)$ and $\varepsilon_{\underline{R}}^{A}(1)$, respectively, for left-up, left-down, right-up and right-down sites, as well as effective hopping integrals ${t^{\prime }}_{h}(1),\;{t^{\prime }}_{\underline{h}}(1),\;{t^{\prime }}_{v}(1),\;{t^{\prime }}_{\underline{v}}(1),\;{t^{\prime }}_{d}(1)$ and ${t^{\prime }}_{\underline{d}}(1)$, correspondingly denoting up horizontal, down horizontal, left vertical, right vertical, decreasing diagonal and increasing diagonal bonds. Using the addition rule for *A*-type RS dual channels given by Equation (A21) and Table A2, this four sites dual channel is concatenated to another four-site renormalized *B*-type RS dual channel to build a six-site dual channel of generation 2, where the on-site energies of central sites are $\varepsilon_{C}^{A}(2)=\varepsilon_{R}^{A}(1)+\varepsilon_{L}^{B}(1)-\varepsilon$ and $\varepsilon_{\underline{C}}^{A}(2)=\varepsilon_{\underline{R}}^{A}(1)+\varepsilon_{\underline{L}}^{B}(1)+\varepsilon$ respectively for up and down ones connected by the hopping integral $t_{C}^{A}(2)=t_{\underline{v}}^{A}(1)+t_{v}^{B}(1)$, represented by a double line.

In general, for an α-type RS dual channel of generation *k*, similar addition and renormalization procedures were carried out. The effective on-site energies $\varepsilon_{l}^{\alpha }(k,E)$ with $l=L,\;\underline{L},\;R\;\mathrm{or}\;\underline{R}$ and hopping integrals $t_{j}^{\alpha }(k,E)$, being $l=h,\;\underline{h},\;v,\;\underline{v},\;d\;\mathrm{or}\;\underline{d}$, of four-site dual channel can be obtained from the stationary Schrödinger equation:

(A22) $$(H-EI)|\Psi \rangle =0$$

where **I** is the identity matrix, **H** is the Hamiltonian of a concatenated six-site dual channel built from two dual channels of generations k−1 and $|\Psi \rangle =\sum_{j}b_{j}|j\rangle$ is the electronic wavefunction with $j=L,\;\underline{L},\;C,\;\underline{C},\;R,\;\mathrm{or}\;\underline{R}$. Equation (A22) can be rewritten as

(A23) $$\left(\begin{array}{cccccc} \varepsilon_{L}^{\beta }\left(k-1,E\right)-E & t_{v}^{\beta }\left(k-1,E\right) & t_{h}^{\beta }\left(k-1,E\right) & t_{d}^{\beta }\left(k-1,E\right) & 0 & 0\\ t_{v}^{\beta }\left(k-1,E\right) & \varepsilon_{\underline{L}}^{\beta }\left(k-1,E\right)-E & t_{\underline{d}}^{\beta }\left(k-1,E\right) & t_{\underline{h}}^{\beta }\left(k-1,E\right) & 0 & 0\\ t_{h}^{\beta }\left(k-1,E\right) & t_{\underline{d}}^{\beta }\left(k-1,E\right) & \varepsilon_{C}^{\alpha }\left(k,E\right)-E & t_{c}^{\alpha }\left(k,E\right) & t_{\underline{h}}^{\gamma }\left(k-1,E\right) & t_{d}^{\gamma }\left(k-1,E\right)\\ t_{d}^{\beta }\left(k-1,E\right) & t_{\underline{h}}^{\beta }\left(k-1,E\right) & t_{c}^{\alpha }\left(k,E\right) & \varepsilon_{\underline{C}}^{\alpha }\left(k,E\right)-E & t_{\underline{d}}^{\gamma }\left(k-1,E\right) & t_{\underline{h}}^{\gamma }\left(k-1,E\right)\\ 0 & 0 & t_{h}^{\gamma }\left(k-1,E\right) & t_{\underline{d}}^{\gamma }\left(k-1,E\right) & \varepsilon_{R}^{\gamma }\left(k-1,E\right)-E & t_{\underline{v}}^{\gamma }\left(k-1,E\right)\\ 0 & 0 & t_{d}^{\gamma }\left(k-1,E\right) & t_{\underline{h}}^{\gamma }\left(k-1,E\right) & t_{\underline{v}}^{\gamma }\left(k-1,E\right) & \varepsilon_{\underline{R}}^{\gamma }\left(k-1,E\right)-E \end{array}\right)\left(\begin{array}{c} b_{L}^{\beta }\\ b_{\underline{L}}^{\beta }\\ b_{C}^{\alpha }\\ b_{\underline{C}}^{\alpha }\\ b_{R}^{\gamma }\\ b_{\underline{R}}^{\gamma } \end{array}\right)=(\begin{array}{c} 0\\ 0\\ 0\\ 0\\ 0\\ 0 \end{array}),$$

whose two central equations can be considered as a set of equations for $b_{C}^{\alpha }$ and $b_{\underline{C}}^{\alpha }$, with solutions given by

(A24) $$\left\{\begin{array}{c} b_{C}^{\alpha }=\Gamma_{1}^{\alpha }(k,E)\;b_{L}^{\beta }+\Gamma_{2}^{\alpha }(k,E)\;b_{\underline{L}}^{\beta }+\Gamma_{3}^{\alpha }(k,E)\;b_{R}^{\gamma }+\Gamma_{4}^{\alpha }(k,E)\;b_{\underline{R}}^{\gamma }\\ b_{\underline{C}}^{\alpha }=\Theta_{1}^{\alpha }(k,E)\;b_{L}^{\beta }+\Theta_{2}^{\alpha }(k,E)\;b_{\underline{L}}^{\beta }+\Theta_{3}^{\alpha }(k,E)\;b_{R}^{\gamma }+\Theta_{4}^{\alpha }(k,E)\;b_{\underline{R}}^{\gamma } \end{array}\right.,$$

where

(A25) $$\left\{\begin{array}{c} \Gamma_{1}^{\alpha }(k,E)=\theta_{1}^{\alpha }(k,E)\;[t_{h}^{\beta }(k-\;1,E)\;+\;\phi_{2}^{\alpha }(k,E)\;t_{d}^{\beta }(k-\;1,E)]\\ \Gamma_{2}^{\alpha }(k,E)=\theta_{1}^{\alpha }(k,E)\;[t_{\underline{d}}^{\beta }(k-\;1,E)\;+\;\phi_{2}^{\alpha }(k,E)\;t_{\underline{h}}^{\beta }(k-\;1,E)]\\ \Gamma_{3}^{\alpha }(k,E)=\theta_{1}^{\alpha }(k,E)\;[t_{h}^{\gamma }(k-1,E)+\phi_{2}^{\alpha }(k,E)\;t_{\underline{d}}^{\gamma }(k-1,E)]\\ \Gamma_{4}^{\alpha }(k,E)=\theta_{1}^{\alpha }(k,E)\;[t_{d}^{\gamma }(k-1,E)+\phi_{2}^{\alpha }(k,E)\;t_{\underline{h}}^{\gamma }(k-1,E)] \end{array}\right.$$

and

(A26) $$\left\{\begin{array}{c} \Theta_{1}^{\alpha }(k,E)=\theta_{2}^{\alpha }(k,E)\;[t_{d}^{\beta }(k-1,E)+\phi_{1}^{\alpha }(k,E)\;t_{h}^{\beta }(k-1,E)]\\ \Theta_{2}^{\alpha }(k,E)=\theta_{2}^{\alpha }(k,E)\;[t_{\underline{h}}^{\beta }(k-1,E)+\phi_{1}^{\alpha }(k,E)\;t_{\underline{d}}^{\beta }(k-1,E)]\\ \Theta_{3}^{\alpha }(k,E)=\theta_{2}^{\alpha }(k,E)\;[t_{\underline{d}}^{\gamma }(k-1,E)+\phi_{1}^{\alpha }(k,E)\;t_{h}^{\gamma }(k-1,E)]\\ \Theta_{4}^{\alpha }(k,E)=\theta_{2}^{\alpha }(k,E)\;[t_{\underline{h}}^{\gamma }(k-1,E)+\phi_{1}^{\alpha }(k,E)\;t_{d}^{\gamma }(k-1,E)] \end{array}\right.,$$

where

(A27) $$\left\{\begin{array}{c} \theta_{1}^{\alpha }(k,E)={\left\{E-\varepsilon_{c}^{\alpha }(k,E)-\frac{{\left[t_{c}^{\alpha }(k,E)\right]}^{2}}{E-\varepsilon_{\underline{c}}^{\alpha }(k,E)}\right\}}^{-1}\\ \theta_{2}^{\alpha }(k,E)={\left\{E-\varepsilon_{\underline{c}}^{\alpha }(k,E)-\frac{{\left[t_{c}^{\alpha }(k,E)\right]}^{2}}{E-\varepsilon_{c}^{\alpha }(k,E)}\right\}}^{-1} \end{array}\right.\;\mathrm{and}\;\left\{\begin{array}{c} \phi_{1}^{\alpha }(k,E)=\frac{t_{c}^{\alpha }(k,E)}{E-\varepsilon_{c}^{\alpha }(k,E)}\\ \phi_{2}^{\alpha }(k,E)=\frac{t_{c}^{\alpha }(k,E)}{E-\varepsilon_{\underline{c}}^{\alpha }(k,E)} \end{array}\right.$$

with

(A28) $$\left\{\begin{array}{c} \varepsilon_{c}^{\alpha }(k,E)=\varepsilon_{R}^{\beta }(k-1,E)+\varepsilon_{L}^{\gamma }(k-1,E)-\varepsilon \\ \varepsilon_{\underline{c}}^{\alpha }(k,E)=\varepsilon_{\underline{R}}^{\beta }(k-1,E)+\varepsilon_{\underline{L}}^{\gamma }(k-1,E)+\varepsilon \\ t_{c}^{\alpha }(k,E)=t_{\underline{v}}^{\beta }(k-1,E)+t_{v}^{\gamma }(k-1,E) \end{array}\right..$$

Substituting Equation (A24) into (A23), we obtain the stationary Schrödinger equation of a four-site dual channel given by

(A29) $$\left(\begin{array}{cccc} \varepsilon_{L}^{\alpha }(k,E)-E & t_{v}^{\alpha }(k,E) & t_{h}^{\alpha }(k,E) & t_{d}^{\alpha }(k,E)\\ t_{v}^{\alpha }(k,E) & \varepsilon_{\underline{L}}^{\alpha }(k,E)-E & t_{\underline{d}}^{\alpha }(k,E) & t_{\underline{h}}^{\alpha }(k,E)\\ t_{h}^{\alpha }(k,E) & t_{\underline{d}}^{\alpha }(k,E) & \varepsilon_{R}^{\alpha }(k,E)-E & t_{\underline{v}}^{\alpha }(k,E)\\ t_{d}^{\alpha }(k,E) & t_{\underline{h}}^{\alpha }(k,E) & t_{\underline{v}}^{\alpha }(k,E) & \varepsilon_{\underline{R}}^{\alpha }(k,E)-E \end{array}\right)\left(\begin{array}{c} b_{L}^{\alpha }\\ b_{\underline{L}}^{\alpha }\\ b_{R}^{\alpha }\\ b_{\underline{R}}^{\alpha } \end{array}\right)=\left(\begin{array}{c} 0\\ 0\\ 0\\ 0 \end{array}\right),$$

where

(A30) $$\left\{ \begin{array}{c} \varepsilon_{L}^{\alpha }(k,E)=\varepsilon_{L}^{\beta }(k-\;1,E)+\Gamma_{1}^{\alpha }(k,E)\;t_{h}^{\beta }(k-\;1,E)+\Theta_{1}^{\alpha }(k,E)\;t_{d}^{\beta }(k-1,E)\\ \varepsilon_{\underline{L}}^{\alpha }(k,E)=\varepsilon_{\underline{L}}^{\beta }(k-\;1,E)+\Theta_{2}^{\alpha }(k,E)\;t_{\underline{h}}^{\beta }(k-\;1,E)+\Gamma_{2}^{\alpha }(k,E)\;t_{\underline{d}}^{\beta }(k-1,E)\\ \varepsilon_{R}^{\alpha }(k,E)=\varepsilon_{R}^{\gamma }(k-\;1,E)+\Gamma_{3}^{\alpha }(k,E)\;t_{h}^{\gamma }(k-\;1,E)+\Theta_{3}^{\alpha }(k,E)\;t_{\underline{d}}^{\gamma }(k-1,E)\\ \varepsilon_{\underline{R}}^{\alpha }(k,E)=\varepsilon_{\underline{R}}^{\gamma }(k-\;1,E)+\Theta_{4}^{\alpha }(k,E)\;t_{\underline{h}}^{\gamma }(k-\;1,E)+\Gamma_{4}^{\alpha }(k,E)\;t_{d}^{\gamma }(k-1,E) \end{array}\right.$$

and

(A31) $$\left\{ \begin{array}{c} t_{h}^{\alpha }(k,E)=\Gamma_{3}^{\alpha }(k,E)\;t_{h}^{\beta }(k-1,E)+\Theta_{3}^{\alpha }(k,E)\;t_{d}^{\beta }(k-1,E)\\ t_{\underline{h}}^{\alpha }(k,E)=\Gamma_{4}^{\alpha }(k,E)\;t_{\underline{d}}^{\beta }(k-1,E)+\Theta_{4}^{\alpha }(k,E)\;t_{\underline{h}}^{\beta }(k-1,E)\\ t_{d}^{\alpha }(k,E)=\Gamma_{4}^{\alpha }(k,E)\;t_{h}^{\beta }(k-1,E)+\Theta_{4}^{\alpha }(k,E)\;t_{d}^{\beta }(k-1,E)\\ t_{\underline{d}}^{\alpha }(k,E)=\Gamma_{3}^{\alpha }(k,E)\;t_{\underline{d}}^{\beta }(k-1,E)+\Theta_{3}^{\alpha }(k,E)\;t_{\underline{h}}^{\beta }(k-1,E)\\ t_{v}^{\alpha }(k,E)=t_{v}^{\beta }(k-1,E)+\Gamma_{2}^{\alpha }(k,E)\;t_{h}^{\beta }(k-1,E)+\Theta_{2}^{\alpha }(k,E)\;t_{d}^{\beta }(k-1,E)\\ t_{\underline{v}}^{\alpha }(k,E)=t_{\underline{v}}^{\gamma }(k-1,E)+\Gamma_{4}^{\alpha }(k,E)\;t_{h}^{\gamma }(k-1,E)+\Theta_{4}^{\alpha }(k,E)\;t_{\underline{d}}^{\gamma }(k-1,E). \end{array}\right.$$

These effective on-site energies and hopping integrals are those indicated in Figure A5.

On the other hand, the electronic density of states (*DOS*) for an *α*-type RS dual channel of generation *k* evaluated at energy *E* can be written as [22]

(A32) DOSα(k,E)=−1πlimη→0+∑j=1L(k)Im [Gj,jα(z)],

where $L(k)=2\;(1+2^{k})$ is the number of sites in the dual channel and $G_{j,j}^{\alpha }(z)$ is the *j*-th diagonal element of retarded Green’s function evaluated at z=E+iη. Extending the real-space renormalization method developed for Fibonacci chains [37,38], the $DOS^{\alpha }(k,E)$ of (A32) can be rewritten as

(A33) DOSα(k,E)=−1πlimη→0+Im [Mα(k,z) GL,Lα(k,z) +Pα(k,z) GL_,L_α(k,z) + Qα(k,z) GR,Ra(k,z)    +Rα(k,z) GR_,R_α(k,z)+Sα(k,z) GL,L_α(k,z)+Uα(k,z) GL,Rα(k,z)+Vα(k,z) GL,R_α(k,z)  +Wα(k,z) GL_,Rα(k,z)+Xα(k,z) GL_,R_α(k,z)+Yα(k,z) GR,R_α(k,z)+Zα(k,z)],

where $M^{\alpha }(k,z),\;\;\cdots ,\;Z^{\alpha }(k,z)$ are the renormalization coefficients and $G_{l,j}^{\alpha }(k,z)$ with $l,j=L,\;\underline{L},\;R\;\mathrm{or}\;\underline{R}$ are elements of the Green’s function matrix, with $L(\underline{L})$ and $R(\underline{R})$ respectively being the up (down) left and right sites of the renormalized four-site dual channel, as illustrated in Figure A5. From Equation (A32) and the addition rule (A21), we have

(A34) $$DOS^{\alpha }(k,E)=DOS^{\beta }(k-1,E)+DOS^{\gamma }(k-1,E)-G_{C,C}^{\alpha }(k,z)-G_{\underline{C},\underline{C}}^{\alpha }(k,z),$$

where subindex *C* denotes the central sites at the union of two renormalized dual channels. Equation (A34) can be rewritten using Equation (A33) as

(A35) DOSα(k,E)=−1πlimη→0+Im [Mβ(k−1,z)GL,Lβ(k,z)+Pβ(k−1,z)GL_,L_β(k,z)+Qβ(k−1,z)GC,Cβ(k,z) +Rβ(k−1,z)GC_,C_β(k,z)+Sβ(k−1,z)GL,L_β(k,z)+Uβ(k−1,z)GL,Cβ(k,z)+Vβ(k−1,z)GL,C_β(k,z) +Wβ(k−1,z)GL_,Cβ(k,z)+Xβ(k−1,z)GL_,C_β(k,z)+Yβ(k−1,z)GC,C_β(k,z)+Zβ(k−1,z) +Mγ(k−1,z)GC,Cγ(k,z)+Pγ(k−1,z)GC_,C_γ(k,z)+Qγ(k−1,z)GR,Rγ(k,z)+Rγ(k−1,z)GR_,R_γ(k,z) +Sγ(k−1,z)GC,C_γ(k,z)+Uγ(k−1,z)GC,Rγ(k,z)+Vγ(k−1,z)GC,R_γ(k,z)+Wγ(k−1,z)GC_,Rγ(k,z) +Xγ(k−1,z)GC_,R_γ(k,z)+Yγ(k−1,z)GR,R_γ(k,z)+Zγ(k−1,z)−GC,Cγ(k,z)−GC_,C_γ(k,z)].

The Dyson equation of a six-site *α*-type RS dual channel (see Figure A5 for α=A) given by (zI−H)G=I can be rewritten as

(A36) $$\left\{ \begin{array}{c} \left[z-\varepsilon_{L}^{\beta }(k-1)]G_{L,j}^{\beta }(k-1,z)=\delta_{L,j}+t_{v}^{\beta }(k-1)G_{\underline{L},j}^{\beta }(k-1,z)+t_{h}^{\beta }(k-1)G_{C,j}^{\alpha }(k,z)+t_{d}^{\beta }(k-1)G_{\underline{C},j}^{\alpha }(k,z\right)\\ {}[z-\varepsilon_{\underline{L}}^{\beta }(k-1)]G_{\underline{L},j}^{\beta }(k-1,z)=\delta_{\underline{L},j}+t_{v}^{\beta }(k-1)G_{L,j}^{\beta }(k-1,z)+t_{\underline{d}}^{\beta }(k-1)G_{C,j}^{\alpha }(k,z)+t_{\underline{h}}^{\beta }(k-1)G_{\underline{C},j}^{\alpha }(k,z)\;\\ \left[z-\varepsilon_{C}^{\alpha }(k)]G_{C,j}^{\alpha }(k,z)=\delta_{C,j}+t_{h}^{\beta }(k-1)G_{L,j}^{\beta }(k-1,z)+t_{\underline{d}}^{\beta }(k-1)G_{\underline{L},j}^{\beta }(k-1,z)+t_{\underline{h}}^{\gamma }(k-1)G_{R,j}^{\gamma }(k-1,z\right)\\ +t_{d}^{\gamma }(k-1)G_{\underline{R},j}^{\gamma }(k-1,z)+t_{c}^{\alpha }(k)G_{\underline{C},j}^{\alpha }(k,z)\\ \left[z-\varepsilon_{\underline{C}}^{\alpha }(k)]G_{\underline{C},j}^{\alpha }(k,z)=\delta_{\underline{C},j}+t_{d}^{\beta }(k-1)G_{L,j}^{\beta }(k-1,z)+t_{\underline{h}}^{\beta }(k-1)G_{\underline{L},j}^{\beta }(k-1,z)+t_{\underline{d}}^{\gamma }(k-1)G_{R,j}^{\gamma }(k-1,z\right)\\ +t_{\underline{h}}^{\gamma }(k-1)G_{\underline{R},j}^{\gamma }(k-1,z)+t_{c}^{\alpha }(k)G_{C,j}^{\alpha }(k,z)\\ \left[z-\varepsilon_{R}^{\gamma }(k-1)]G_{R,j}^{\beta }(k-1,z)=\delta_{R,j}+t_{\underline{v}}^{\gamma }(k-1)G_{\underline{R},j}^{\beta }(k-1,z)+t_{h}^{\gamma }(k-1)G_{C,j}^{\alpha }(k,z)+t_{\underline{d}}^{\gamma }(k-1)G_{\underline{C},j}^{\alpha }(k,z\right)\\ \left[z-\varepsilon_{\underline{R}}^{\gamma }(k-1)]G_{\underline{R},j}^{\beta }(k-1,z)=\delta_{\underline{R},j}+t_{\underline{v}}^{\gamma }(k-1)G_{R,j}^{\beta }(k-1,z)+t_{d}^{\gamma }(k-1)G_{C,j}^{\alpha }(k,z)+t_{\underline{h}}^{\gamma }(k-1)G_{\underline{C},j}^{\alpha }(k,z\right) \end{array}\right.$$

where $j=L,\;\underline{L},\;C,\;\underline{C},\;R,\;\mathrm{or}\;\underline{R}$, and then Equation (A36) represents the 36 equations of six effective sites. The two central equations of (A36) can be visualized as a system of equations for $G_{C,j}^{\alpha }(k,z)$ and $G_{\underline{C},j}^{\alpha }(k,z)$, whose solutions are

(A37) $$\left\{ \begin{array}{c} G_{C,j}^{\alpha }(k,z)=\Gamma_{1}^{\alpha }(k,z)\;G_{L,j}^{\beta }(k-1,z)+\Gamma_{2}^{\alpha }(k,z)\;G_{\underline{L},j}^{\beta }(k-1,z)+\Gamma_{3}^{\alpha }(k,z)\;G_{R,j}^{\gamma }(k-1,z)\\ +\Gamma_{4}^{\alpha }(k,z)\;G_{\underline{R},j}^{\gamma }(k-1,z)+\delta_{C,j}+\theta_{1}^{\alpha }(k,z)\delta_{\underline{C},j}\\ G_{\underline{C},j}^{\alpha }(k,z)=\Theta_{1}^{\alpha }(k,z)\;G_{L,j}^{\beta }(k-1,z)+\Theta_{2}^{\alpha }(k,z)\;G_{\underline{L},j}^{\beta }(k-1,z)+\Theta_{3}^{\alpha }(k,z)\;G_{R,j}^{\gamma }(k-1,z)\\ +\Theta_{4}^{\alpha }(k,z)\;G_{\underline{R},j}^{\gamma }(k-1,z)+\delta_{\underline{C},j}+\theta_{2}^{\alpha }(k,z)\delta_{C,j} \end{array}.\right.$$

Substituting Equation (A37) into (A35) and comparing it to Equation (A33), we obtain the following iteration relations for renormalization coefficients given by

(A38) $$M^{\alpha }(k,z)\;=M^{\beta }(k-1,z)+\Gamma_{1}^{\alpha }(k,z)\;[\Xi_{1}^{\alpha }(k,z)+U^{\beta }(k-1,z)]+\Theta_{1}^{\alpha }(k,z)\;[V^{\beta }(k-1,z)+\Omega_{1}^{\alpha }(k,z)]$$

(A39) $$P^{\alpha }(k,z)\;=\;P^{\beta }(k-1,z)+\Gamma_{2}^{\alpha }(k,z)\;[\Xi_{2}^{\alpha }(k,z)+W^{\beta }(k-1,z)]+\Theta_{2}^{\alpha }(k,z)\;[X^{\beta }(k-1,z)+\Omega_{2}^{\alpha }(k,z)],$$

(A40) $$Q^{\alpha }(k,z)\;=Q^{\gamma }(k-1,z)+\Gamma_{3}^{\alpha }(k,z)\;[\Xi_{3}^{\alpha }(k,z)+U^{\gamma }(k-1,z)]+\Theta_{3}^{\alpha }(k,z)\;[W^{\gamma }(k-1,z)+\Omega_{3}^{\alpha }(k,z)],$$

(A41) $$R^{\alpha }(k,z)=\;R^{\gamma }(k-1,z)+\Gamma_{4}^{\alpha }(k,z)\;[\Xi_{4}^{\alpha }(k,z)+V^{\gamma }(k-1,z)]+\Theta_{4}^{\alpha }(k,z)\;[X^{\gamma }(k-1,z)+\Omega_{4}^{\alpha }(k,z)],$$

(A42) $$\begin{array}{c} S^{\alpha }(k,z)=S^{\beta }(k-1,z)+\Gamma_{2}^{\alpha }(k,z)\;[\Xi_{1}^{\alpha }(k,z)+U^{\beta }(k-1,z)]+\Gamma_{1}^{\alpha }(k,z)\;[\Xi_{2}^{\alpha }(k,z)+W^{\beta }(k-1,z)]\\ \;\;\;\;+\Theta_{2}^{\alpha }(k,z)\;[V^{\beta }(k-1,z)+\Omega_{1}^{\alpha }(k,z)]+\Theta_{1}^{\alpha }(k,z)\;[X^{\beta }(k-1,z)+\Omega_{2}^{\alpha }(k,z)], \end{array}$$

(A43) $$\begin{array}{c} U^{\alpha }(k,z)=\Gamma_{3}^{\alpha }(k,z)\;[\Xi_{1}^{\alpha }(k,z)+U^{\beta }(k-1,z)]+\Gamma_{1}^{\alpha }(k,z)\;[\Xi_{3}^{\alpha }(k,z)+U^{\gamma }(k-1,z)]\\ \;\;\;\;+\Theta_{3}^{\alpha }(k,z)\;[V^{\beta }(k-1,z)+\Omega_{1}^{\alpha }(k,z)]+\Theta_{1}^{\alpha }(k,z)\;[W^{\gamma }(k-1,z)+\Omega_{3}^{\alpha }(k,z)], \end{array}$$

(A44) $$\begin{array}{c} V^{\alpha }(k,z)=\Gamma_{4}^{\alpha }(k,z)\;[\Xi_{1}^{\alpha }(k,z)+U^{\beta }(k-1,z)]+\Gamma_{1}^{\alpha }(k,z)\;[\Xi_{4}^{\alpha }(k,z)+V^{\gamma }(k-1,z)]\\ \;\;\;\;+\Theta_{4}^{\alpha }(k,z)\;[V^{\beta }(k-1,z)+\Omega_{1}^{\alpha }(k,z)]+\Theta_{1}^{\alpha }(k,z)\;[X^{\gamma }(k-1,z)+\Omega_{4}^{\alpha }(k,z)], \end{array}$$

(A45) $$\begin{array}{c} W^{\alpha }(k,z)=\Gamma_{3}^{\alpha }(k,z)\;[\Xi_{2}^{\alpha }(k,z)+W^{\beta }(k-1,z)]+\Gamma_{2}^{\alpha }(k,z)\;[\Xi_{3}^{\alpha }(k,z)+U^{\gamma }(k-1,z)]\\ \;\;\;\;+\Theta_{3}^{\alpha }(k,z)\;[X^{\beta }(k-1,z)+\Omega_{2}^{\alpha }(k,z)]+\Theta_{2}^{\alpha }(k,z)\;[W^{\gamma }(k-1,z)+\Omega_{3}^{\alpha }(k,z)], \end{array}$$

(A46) $$\begin{array}{c} X^{\alpha }(k,z)=\Gamma_{4}^{\alpha }(k,z)\;[\Xi_{2}^{\alpha }(k,z)+W^{\beta }(k-1,z)]+\Gamma_{2}^{\alpha }(k,z)\;[\Xi_{4}^{\alpha }(k,z)+V^{\gamma }(k-1,z)]\\ \;\;\;\;+\Theta_{4}^{\alpha }(k,z)\;[X^{\beta }(k-1,z)+\Omega_{2}^{\alpha }(k,z)]+\Theta_{2}^{\alpha }(k,z)\;[X^{\gamma }(k-1,z)+\Omega_{4}^{\alpha }(k,z)], \end{array}$$

(A47) $$\begin{array}{c} Y^{\alpha }(k,z)=\;Y^{\gamma }(k-1,z)+\Gamma_{4}^{\alpha }(k,z)\;[\Xi_{3}^{\alpha }(k,z)+U^{\gamma }(k-1,z)]+\Gamma_{3}^{\alpha }(k,z)\;[\Xi_{4}^{\alpha }(k,z)+V^{\gamma }(k-1,z)]\\ \;\;\;\;+\Theta_{4}^{\alpha }(k,z)\;[W^{\gamma }(k-1,z)+\Omega_{3}^{\alpha }(k,z)]+\Theta_{3}^{\alpha }(k,z)\;[X^{\gamma }(k-1,z)+\Omega_{4}^{\alpha }(k,z)], \end{array}$$

(A48) $$\begin{array}{c} Z^{\alpha }(k,z)=Z^{\beta }(k-1,z)+Z^{\gamma }(k-1,z)+\theta_{1}^{\alpha }(k,z)\;[Q^{\beta }(k-1,z)+M^{\gamma }(k-1,z)-1]\\ \;\;\;\;+\theta_{2}^{\alpha }(k,z)\;[R^{\beta }(k-1,z)+P^{\gamma }(k-1,z)-1]+\phi_{2}^{\alpha }(k,z)\;[Y^{\beta }(k-1,z)+S^{\gamma }(k-1,z)], \end{array}$$

with

(A49) $$\Xi_{\rho }^{\alpha }(k,z)=\Gamma_{\rho }^{\alpha }(k,z)\;[Q^{\beta }(k-1,z)+M^{\gamma }(k-1,z)-1]+\Theta_{\rho }^{\alpha }(k,z)\;[Y^{\beta }(k-1,z)+S^{\gamma }(k-1,z)]$$

and

(A50) $$\Omega_{\rho }^{\alpha }(k,z)\;=\;\Theta_{\rho }^{\alpha }(k,z)\;[R^{\beta }(k-1,z)+P^{\gamma }(k-1,z)-1],$$

where ρ=1, 2, 3 or 4. The initial conditions for this renormalization method are

(A51) $$\left\{\begin{array}{c} M^{\alpha }(1,z)=P^{\alpha }(1,z)=Q^{\alpha }(1,z)=R^{\alpha }(1,z)\;=1+(\xi_{-}^{2}+\xi_{+}^{2})\;t_{\alpha }^{2}\\ U^{\alpha }(1,z)\;=-V^{\alpha }(1,z)=W^{\alpha }(1,z)=-X^{\alpha }(1,z)=2(\xi_{-}^{2}-\xi_{+}^{2})\;t_{\alpha }^{2}\\ S^{\alpha }(1,z)\;=-Y^{\alpha }(1,z)=2(\xi_{-}^{2}+\xi_{+}^{2})\;t_{\alpha }^{2}\\ Z^{\alpha }(1,z)=(\xi_{-}+\xi_{+}) \end{array}\right.$$

and

(A52) $$\left\{\begin{array}{c} t_{h}^{\alpha }(1,z)=-t_{\underline{h}}^{\alpha }(1,z)=-t_{d}^{\alpha }(1,z)=t_{\underline{d}}^{\alpha }(1,z)=(\xi_{-}-\xi_{+})\;t_{\alpha }^{2}\\ t_{v}^{\alpha }(1,z)=-t_{\underline{v}}^{\alpha }(1,z)=(\xi_{-}+\xi_{+})\;t_{\alpha }^{2}\\ \varepsilon_{L}^{\alpha }(1,z)=\varepsilon_{R}^{\alpha }(1,z)=(\xi_{-}+\xi_{+})\;t_{\alpha }^{2}-\varepsilon \\ \varepsilon_{\underline{L}}^{\alpha }(1,z)=\varepsilon_{\underline{R}}^{\alpha }(1,z)=(\xi_{-}+\xi_{+})\;t_{\alpha }^{2}+\varepsilon \end{array}\right.,$$

where $\xi_{\pm }\;={\left(z\pm \varepsilon \right)}^{-1}$. The elements of Green’s function in Equation (A33) are numerically calculated from the Dyson equation, written as an 8×8 matrix for the dual channel of the *K*-th generation connected to two periodic leads at its ends, as illustrated at the end of Figure A5.
